# UHMWPE-Assisted Melt Strength Enhancement of Recycled PLA for Lightweight Foaming

**DOI:** 10.1021/acspolymersau.5c00154

**Published:** 2025-11-14

**Authors:** Rostislav Vilem, Ondrej Mertlik, Tomas Plachy, Lukas Manas, Tomas Sedlacek

**Affiliations:** † 561989 Centre of Polymer Systems of Tomas Bata University in Zlín, Tř. T. Bati 5678, Zlín 760 01, Czech Republic

**Keywords:** Polylactic acid, UHMWPE, Polymer−polymer composite, Melt strength, Improved foamability, Thermal conductivity, Sustainability

## Abstract

Increased utilization of biodegradable polymers to achieve sustainability and decrease the carbon footprint is connected with a necessity to find an effective way of their reutilization. Polylactic acid (PLA), and generally all biodegradable polymers, undergoes significant degradation during reprocessing cycles, leading to inferior products when compared with the original material. Here, ultrahigh molecular weight polyethylene (UHMWPE) was incorporated as a reinforcing phase to create a polymer–polymer composite capable of restoring and enhancing the melt strength of recycled PLA. While recycled PLA lost melt strength during reprocessing, limiting its foamability, the UHMWPE/PLA composites exhibited up to a 5.3-fold increase in melt strength, enabling stable cell growth and expansion during foaming. The composite foams also showed a substantial reduction in thermal conductivity to 0.019 W m^–1^ K^–1^ and a lower density of 0.217 g cm^–3^. These improvements originate from the unique combination of UHMWPE/PLA composite and filler morphology within the PLA matrix, which reinforces the PLA melt and suppresses cell coalescence. The approach under discussion relies on standard blending and foaming techniques, thus offering a simple and scalable route for the conversion of reprocessed and recycled PLA into high-performance, highly expanded foams. These foams can be used for thermal insulation, protective packaging and lightweight structural applications.

## Graphical abstract

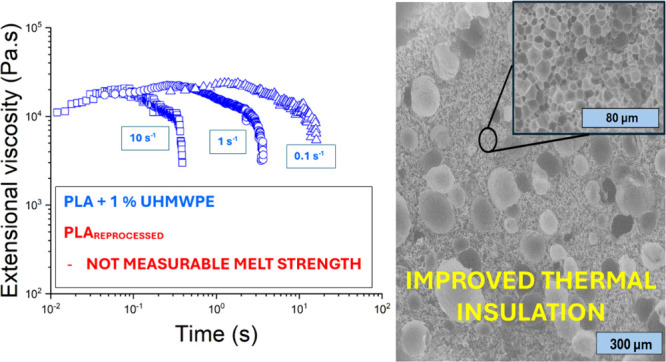

## 1 Introduction

In recent years, substantial efforts have focused on incorporating biodegradable polymers to reduce the environmental impact of plastic materials. These polymers are commonly considered as a sustainable alternative to conventional polymers prepared from oil, since they are often prepared from renewable sources and at the end of their life-cycle, they can be either recycled or transformed to biomass through a biodegradation process. Despite their environmental benefits, biodegradable polymers like polylactic acid (PLA) often suffer from poor melt strength, fragility and limited processing stability, which hinders their broader use.^1,2^ These disadvantages are even promoted during their recycling and reprocessing, leading to their degradation and further inferior polymer material. Romani et al.^3^ reported a 22.7% decrease in shear viscosity after a single reprocessing cycle, and Agüer et al.^4^ presented that the impact strength of neat, injection-molded PLA samples fell from 57.8 kJ·m^–2^ to 30 kJ·m^–2^ after six reprocessing cycles.

Many studies^5,6^ have therefore explored modification and processing routes for biodegradable or biobased polymers to mitigate these limitations. Representative approaches include reinforcement of a PLA matrix with polyethylene terephthalate (PET),^7^ which has increased the impact strength of PLA by up to 7-fold and novel strategy of bulk polymerization reaction of polyvinyl acetate to markedly tune foamability in PLA systems.^8^

In the foaming process, melt strength, referring to the resistance of the material to deformation in the molten state, plays a crucial role during pore growth and subsequent stabilization. A polymer melt with insufficient melt strength may rupture or collapse during expansion, resulting in poorly developed or irregular foams.^9,10^ In addition to intrinsic polymer properties, fillers play a pivotal role in the foaming process, often acting as nucleating agents that facilitate the creation and stabilization of gas bubbles. By providing nucleation sites, fillers control the foam’s cell size, distribution, and density.^11^ Commonly, particles such as talc, silica, natural fibers, polymeric fibers and many others are used as additives improving the foaming process of plastics.^12^ However, the use of ultrahigh molecular weight polyethylene (UHMWPE) as a functional polymeric filler for foaming remains rare. Only a few studies have considered UHMWPE/polypropylene (PP) blends; for example, Chand et al.^13^ examined abrasive-wear behavior in a UHMWPE-filled PP/PET system. Amza et al.^14^ has successfully embedded prefabricated UHMWPE fibers in a PLA matrix in an extrusion 3D printing process. Sufficiently large fibers (0.16 mm) preserved their shape after processing and led to an increase in tensile strength of prepared composite systems, and at the same time, UHMWPE has only a negligible plasticizing effect on the PLA. UHMWPE generally combines high molecular weight, thermal stability, mechanical durability and low surface energy,^15^ making it a suitable candidate to improve the melt strength, particularly under the demanding conditions of polymer foaming. As such, UHMWPE presents a promising functional additive for improving the foamability and structural integrity of PLA-based composite foams. Despite this potential, the application of UHMWPE as a functional filler in PLA foams remains largely underexplored.

This work presents a polymer–polymer composite approach in which UHMWPE is incorporated into recycled PLA to restore melt strength lost during reprocessing and to enable efficient high-expansion foaming. The combination of renewable PLA with UHMWPE reinforcing fillers delivers lightweight composite foams with enhanced functionality while using conventional industrial foaming techniques. This strategy addresses key performance limitations of recycled PLA foams and provides a sustainable pathway for producing high-performance materials with reduced environmental impact.

## 2 Experimental Section

### 2.1 Materials

In order to achieve the objectives of this study, a single PLA material and two distinct types of UHMWPE were selected. The commercially available product Ingeo Biopolymer PLA 2003D, supplied by NatureWorks (USA), which is a transparent general-purpose grade of PLA with a melt flow rate (MFR) of 6 g/10 min (210 °C, 2.16 kg), was utilized as the matrix.

Two types of UHMWPE powders (GUR 4120 and GUR 2126–2; supplied by Celanese Corporation (USA)) were selected to investigate the influence of filler surface treatment on PLA melt properties. GUR 4120 is a standard UHMWPE with an average molecular weight of 4.7 × 10^6^ g/mol and d50 = 120 μm. GUR 2126–2 is a hydrophilic surface-treated UHMWPE with an average molecular weight of 4.2 × 10^6^ g/mol and d50 = 30 μm.

### 2.2 Preparation of Polymer Blends

PLA pellets were dried at 60 °C for 12 h before compounding. An 80/20 wt % PLA/GUR masterbatch was produced on a corotating Maxi26 Compounder LTE26–48 twin-screw extruder (LabTech Engineering Company Ltd., Thailand). The extruder barrel was operated at a temperature range of 110 to 145 °C and a screw speed of 300 rpm. Although this temperature range is below the melting point of PLA2003D, the polymer was successfully melted due to dissipation during processing. Postextrusion steps consisted of a water bath and pelletizer. After compounding, the PLA/GUR blends were dried at 60 °C for 12 h before the subsequent experiments.

### 2.3 Preparation of Testing Specimens

PLA/GUR masterbatches (80/20 wt %) were diluted by mixing with neat PLA granules to achieve final GUR concentrations of 10, 5, 4, 3, 2, and 1 wt % before injection molding. It should be noted that, due to the need to maintain clarity of figures, only four samples (GUR contents of 1–4 wt %) were referenced in the present study. The remaining contents (5–20 wt %) are illustrated in the Supporting Information. Test sheets with dimensions of 110 × 110 × 1 mm were produced using a Mitsubishi 180MET III-15h injection molding machine (Mitsubishi Heavy Industries, Ltd., Japan). The barrel temperature ranged from 180 to 213 °C, while the mold temperature was 35 °C. During injection, an injection pressure of 130 MPa was applied at an injection speed of 20 mm s^–1^. A packing pressure of 75 MPa was maintained for 15 s, followed by a cooling time of 75 s.

### 2.4 Preparation of Polymer Foams

Injection molded sheets with a thickness of 1 mm were cut into 30 × 20 mm specimens and used in batch foaming. The process involved exposing the specimens to a chamber temperature of 190 °C under a nitrogen gas pressure of 10 MPa for 30 min. After saturation, the specimens were cooled to 20 °C while maintaining the pressure to ensure gas retention within the polymer matrix. Subsequently, the foaming was initiated by immersing the samples in an oil bath preheated to 180 °C. The saturation chamber used in this study was self-designed; a technical schematic of the chamber is provided in Supporting Information (Figures S18–20). For a more detailed overview of a comparable foaming methodology, the reader is referred to Richards et al.,^16^ who used a similar batch foaming setup to prepare PLA and PHBV foams. To assess the impact of GUR particles on the PLA foaming process, two reference samples were foamed under the same conditions: (i) a foam prepared from original PLA (further labeled as PLA_0) and (ii) a foam prepared from a “reprocessed PLA”, where the PLA was subjected to the same processing conditions as the PLA/GUR samples (PLA_R).

### 2.5 Scanning Electron Microscopy

The internal morphology (GUR particles) and foam cell structure were investigated by a scanning electron microscope (Phenom Pro, Thermo Fisher Scientific Inc., USA). Individual samples were fractured in liquid nitrogen from strings produced during the compounding process. The characteristics of the GUR particles and foam pores were quantitatively evaluated using NIS-Elements software.

### 2.6 Differential Scanning Calorimetry (DSC)

DSC measurements were conducted using a DSC 1 (Mettler Toledo, Switzerland) to provide a fundamental description of the melting and crystallization behavior of the PLA and PLA/GUR materials. First, thermal history was removed, and subsequent cooling (20 °C min^–1^) and second heating (10 °C min^–1^) were evaluated. The experiments were conducted under a protective nitrogen atmosphere within a temperature range from 25 to 220 °C.

### 2.7 Rheological Measurements

#### 2.7.1 Oscillatory Rheometry

An Anton Paar MCR 502 rheometer (Anton Paar GmbH, Austria) in oscillatory mode with a 1 mm gap between 25 mm parallel discs, with a strain of 1%, guaranteeing a linear viscoelastic region, was used to investigate the viscoelastic behavior of PLA/GUR melts. Measurements included complex viscosity (η*), storage modulus (*G′*) and loss modulus (*G″*) over a frequency range from 600 rad s^–1^ to 0.1 rad s^–1^ at a constant temperature of 200 °C.

#### 2.7.2 Extensional Rheometry

The extensional viscosity of the PLA/GUR melts was determined using the Anton Paar MCR 502 rheometer equipped with the Sentmanat Extensional Rheometer (SER HV-A01). Experiments were conducted at deformation rates of 10, 1, 0.1, and 0.01 s^–1^ at a temperature of 200 °C with a preheating time of 20 s. Specimens were prepared by cutting rectangular samples 20 × 12.5 × 1 mm from dried injection-molded sheets. In accordance with prevailing convention, the evaluation of data was conducted within the initial drum revolution, a practice that serves to minimize the occurrence of edge and wind-up effects.

### 2.8 Thermal Conductivity

A thermal conductivity analyzer (TCi-3-A, C-Therm Technologies Ltd., Canada) was utilized to assess the thermal conductivity of foamed materials. The specimens, with a thickness of 2 mm, were ground to ensure a flat and uniform surface for precise evaluation. The experiment was repeated several times at multiple locations on the specimen, and the total thermal conductivity was then calculated as the average of 20 measurements.

### 2.9 Impact Strength

The impact strength of foamed PLA/GUR samples was evaluated using a Pendulum Impact Tester, Zwick 5113 (ZwickRoell Group, Germany), equipped with a 2.75 J pendulum hammer. The foamed specimens prepared from injection-molded sheets were cut into rectangular samples with dimensions of 40 × 10 × 2 mm. All tests were conducted at laboratory temperature, each repeated ten times per material to ensure statistical reliability.

## 3 Results and Discussion

### 3.1 Morphology of PLA/GUR Masterbatches

Micrographs obtained from scanning electron microscopy (SEM) are presented in Figure 1. The PLA/GUR 4120 masterbatch (untreated UHMWPE) displays ellipsoidal-shaped UHMWPE particles with mean semiaxes of 95 ± 22 μm and 45 ± 8 μm, respectively (Figure 1a). This represents a slight reduction from the original particle size of 130 μm, likely due to deformation from spherical to ellipsoidal shapes caused by high shear and extensional forces during extrusion. As illustrated in Figure 1b, the PLA/GUR 2126–2 sample, which incorporates hydrophilic surface-treated GUR particles, exhibits significantly smaller particles with average semimajor and semiminor diameters of 21 ± 5 μm and 6 ± 2 μm (mean ± standard deviation), respectively - again indicating slight deformation from the original size of 30 μm. For each particle, an ellipse was fitted to the segmented outline, and the major and minor axis lengths were recorded. The average diameters of both materials were calculated from five SEM images, analyzing 150 GUR particles, while the 10 smallest and 10 largest particles were excluded to ensure statistical reliability.

**Figure 1:**
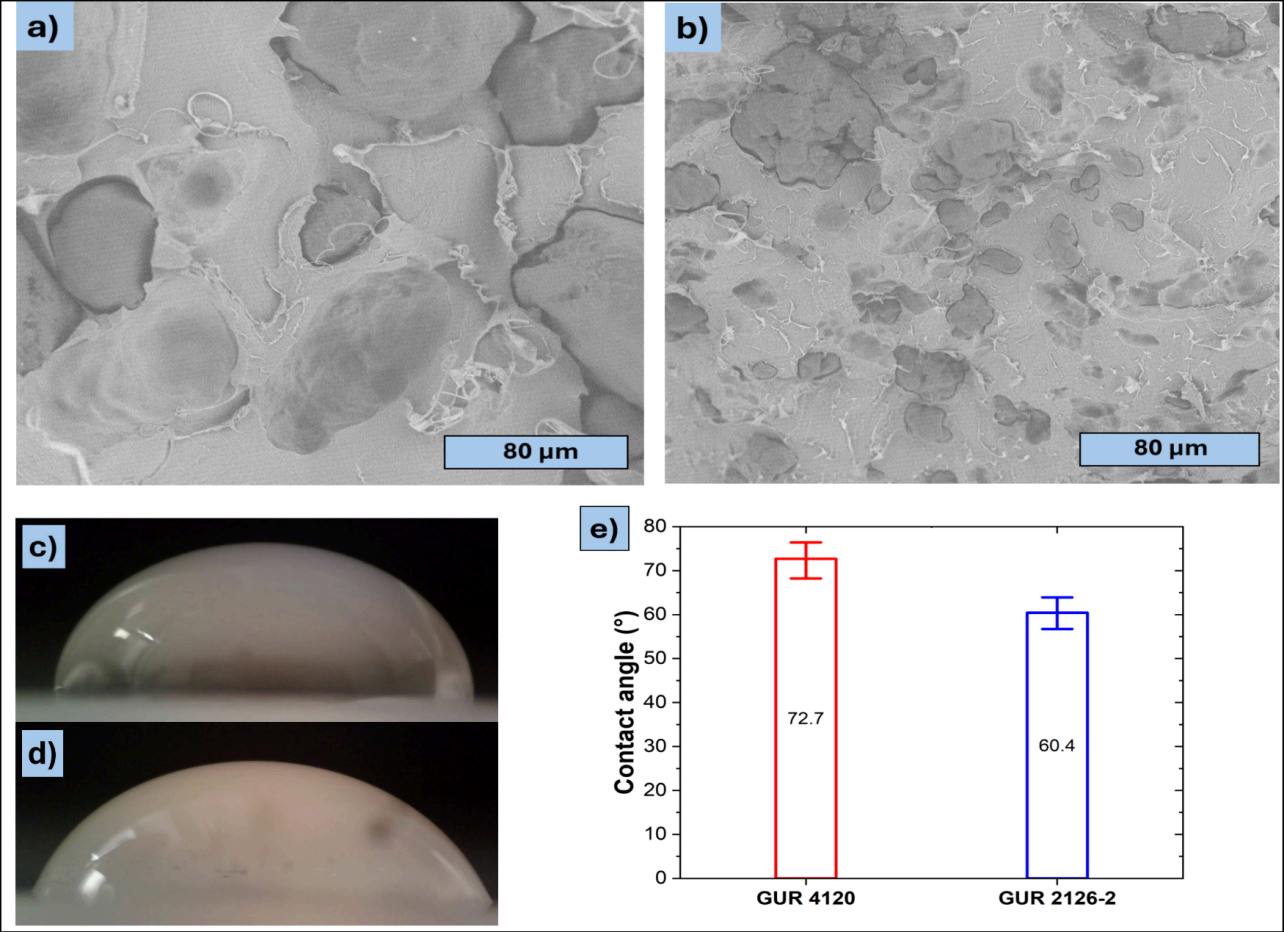
Scanning electron micrographs of PLA/GUR masterbatches: a) PLA/GUR 4120; and b) PLA/GUR 2126–2. c) Sessile water droplet on a GUR 4120 plate; d) Sessile water droplet on a GUR 2126–2 plate; e) Determined static water contact angle for both GUR materials (GUR 4120: untreated; GUR 2126–2: hydrophilically treated). Contact angles present average of 10 droplets (H_2_O, 30 μL). The pictures were taken and contact angle determined using a SEE System 7.0 (Advex Instruments, The Czech Republic).

It is noteworthy that PLA/GUR 4120 shows visible interfacial voids (debonding) around a fraction of UHMWPE particles, whereas PLA/GUR 2126–2 exhibits tighter wetting and fewer gaps, consistent with increased surface energy/adhesion of hydrophilically treated UHMWPE.^17^ In order to verify the occurrence of any change in interfacial tension, the static water contact angles were measured. A comparison of Figure 1c (GUR4120 + H_2_O) and Figure 1d (GUR 2126–2 + H_2_O) reveals a marked decrease in the contact angle for GUR 2126–2, as illustrated in Figure 1e, which provides a quantitative summary of the experimental results. This lower water contact angle indicates a higher (especially polar) component of the UHMWPE surface energy and is consistent with the reduced interfacial voids seen by SEM. While water contact angles do not quantify the PLA–UHMWPE interfacial tension directly, the combined evidence (lower contact angle and fewer interfacial gaps) supports improved interfacial adhesion. Together with the smaller particle size, this is expected to promote morphology-driven rheological reinforcement. For example, Wu et al. reported that foamed PLA/poly­(butylene adipate-*co*-terephthalate (PBAT), surface-modified blend, achieved up to a 9.3-fold increase in impact strength compared with unmodified PLA/PBAT foams.^18^

### 3.2 Morphology of Foams

SEM micrographs of the reference foams (PLA_O, PLA_R), as seen in Figure 2, reveal a uniform pore size distribution in both samples, with no presence of large pores. The primary distinction between the two foams lies in the uniformity of their pore structures; obviously, the reprocessed PLA_R shows a higher fraction of irregular, polygonal cells with sharp edges, consistent with cell-wall rupture and coalescence driven by reduced melt strength after reprocessing.

**Figure 2:**
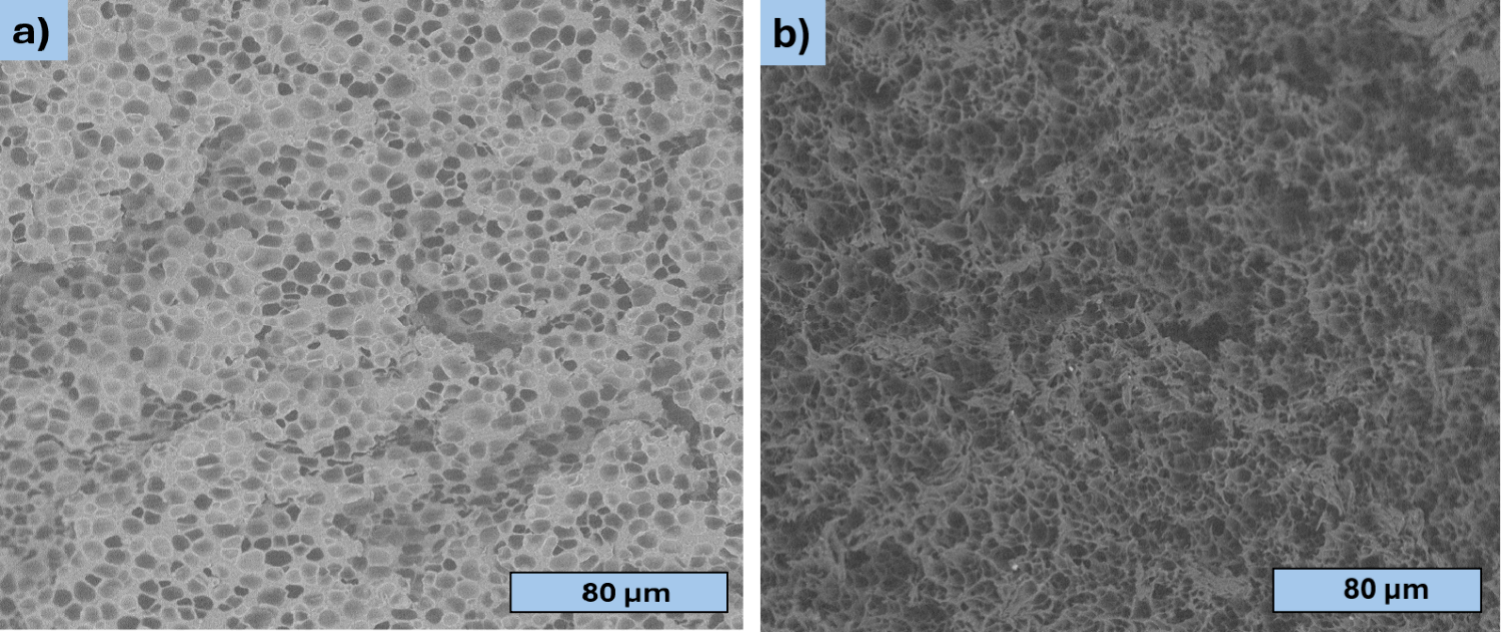
Scanning electron micrographs of PLA foams: a) PLA_O representing original material and b) PLA_R denoting reprocessed material.

SEM micrographs of PLA/GUR foams are shown in Figure 3. Although all concentrations (ranging from 1 to 20 wt %) were successfully foamed, only samples containing 1 wt % GUR particles are displayed here for consistency and clarity. This specific concentration was identified as the most suitable for foam production, as will be discussed in subsequent sections. The key morphological difference between the samples lies in the presence or absence of large pores. While the PLA/GUR 2126–2_1% (hydrophilically treated particles) sample exhibits a bimodal pore structure with a fraction of large pores (Figure 3b), the PLA/GUR 4120_1% (without surface treatment) sample contains only small pores (Figure 3a).

**Figure 3:**
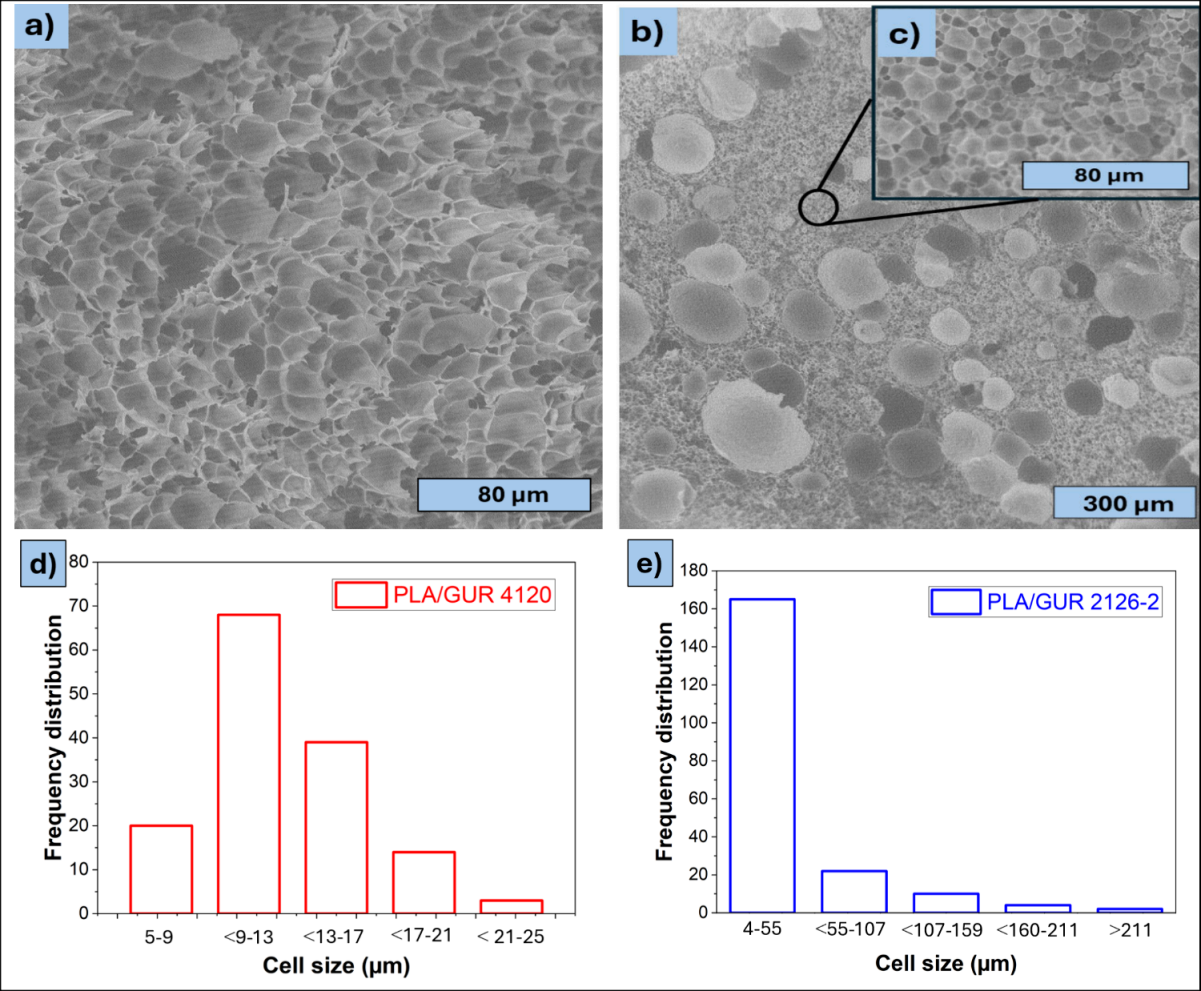
Scanning electron micrographs of PLA/GUR foams with 1 wt % filler content: a) PLA/GUR 4120 sample showing only small pores; b) PLA/GUR 2126–2 sample exhibiting a bimodal pore structure; c) An inset magnifying fraction of smaller pores in PLA/GUR 2126–2 sample; d) Cell size distribution of PLA/GUR 4120_1% foam; e) Cell size distribution of PLA/GUR 2126–2_1% bimodal foam.

This difference in morphology can be attributed to the combined effects of particle size, surface treatment and dispersion of the PLA/GUR 4120 particles within the matrix. In the PLA/GUR 4120_1% sample, the larger untreated GUR particles likely acted as physical barriers that restricted local gas expansion during foaming. Their possible agglomeration may have constrained cell growth, resulting in a structure with only small, isolated pores. Conversely, in the PLA/GUR 2126–2_1% sample, the smaller surface-treated particles created a more heterogeneous nucleation environment. These nucleating sites may have supported the formation of numerous small pores and, under specific local conditions, coalescence or expansion into larger pores, giving rise to a bimodal cell structure (Figure 3d and e). The surface treatment likely enhanced interfacial adhesion and compatibility with the PLA matrix, further improving gas diffusion and pore development. Overall, it is evident that the introduction of GUR particles predominantly contributed to the formation of large pores. When judiciously controlled, bimodal foams can retain or even improve mechanical performance at comparable relative density and are attractive for specific applications, such as acoustic absorption^19^ or filtration.^20^

Since the diameters of tiny pores were comparable across all samples, a direct comparison based on average pore size offers limited insight. The average pore diameter of tiny pores was 9.7 ± 2.8 μm for samples containing untreated GUR particles, and 7.8 ± 2.1 μm for those with surface-treated particles, indicating a slight reduction in pore size with surface treatment. However, to enable a better comparison of foam morphology, the areal fraction of large pores was used as a more relevant comparison criterion (Figure 4). To quantify this criterion, an analyzed region of 1.5 mm^2^ was selected for each sample, and the areal fraction of large pores was subsequently calculated.

**Figure 4:**
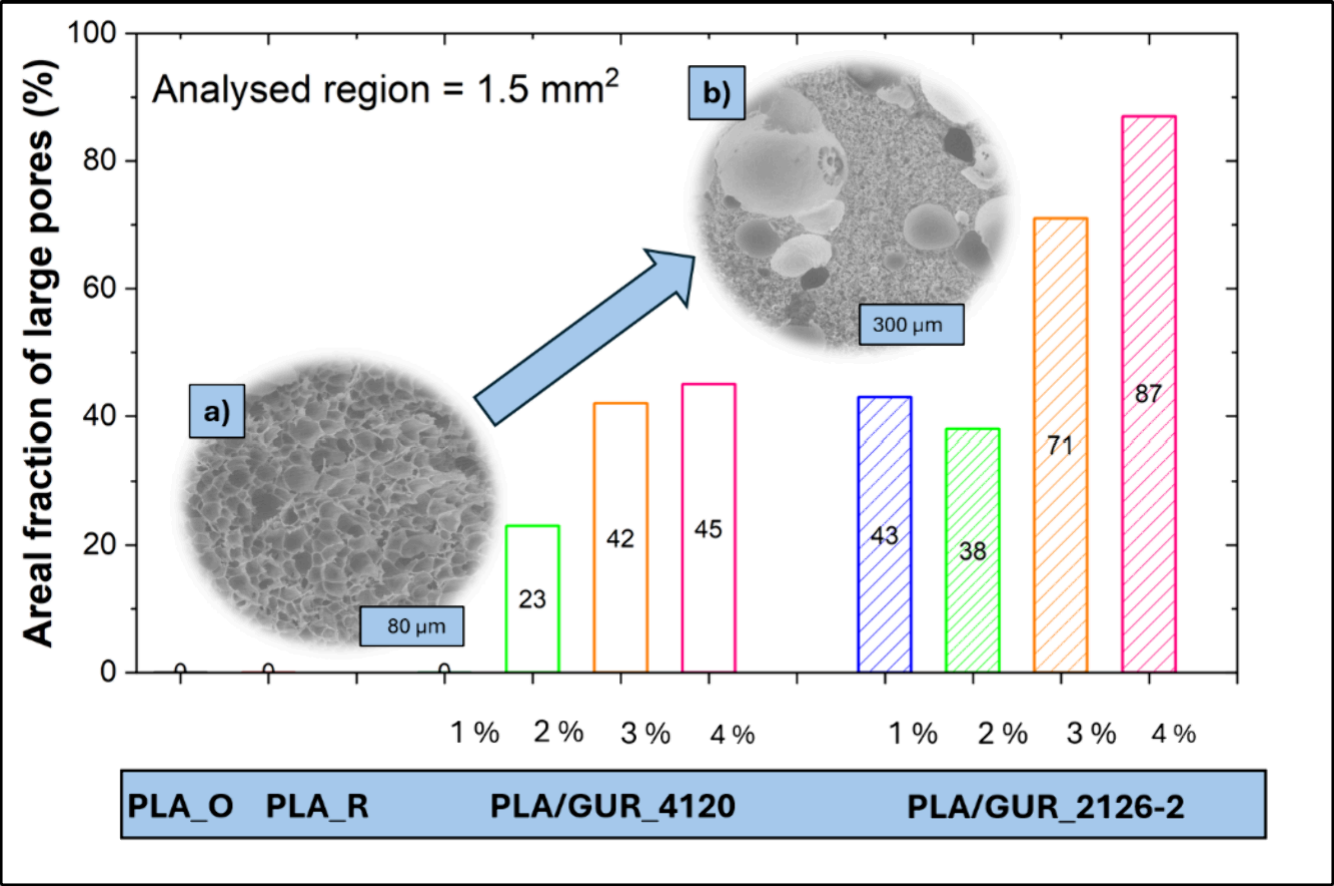
Comparison of the areal fraction of large pores in PLA and PLA/GUR composite foams at four representative filler loadings. The inset (a) shows a SEM image of PLA/GUR 4120_1% and the inset (b) shows a SEM image of PLA/GUR 4120_4%.

The trend observed in Figure 4, where increasing filler content corresponds to a higher fraction of large pores, has been previously reported in the literature.^21^ This phenomenon is primarily attributed to the following mechanisms: (i) agglomeration of fillers at high concentrations disrupts uniform bubble nucleation and promotes the formation of large voids; and (ii) foam collapses due to poor dispersion, as inadequate filler dispersion weakens the foam structure, allowing larger pores to dominate. Agglomeration of fillers at high concentrations disrupts uniform bubble nucleation and promotes the formation of large voids.

Recent studies indicate that bimodal cellular structures (limited fraction of larger cells embedded in a micro/nanocellular matrix), where the fraction and size of the large-cell population are present at certain levels, can maintain or even improve mechanical performance at comparable relative density. In poly­(methyl methacrylate) and PP systems, such bimodal foams achieved lower density while preserving stiffness/strength or markedly enhanced toughness compared with unimodal foams.^22,23^

### 3.3 Differential Scanning Calorimetry

The thermal characteristics of PLA and PLA/GUR samples were investigated with the help of DSC. The primary objective of this analysis was to evaluate the impact of GUR particles on the crystallization behavior of PLA, as crystallization plays a crucial role in determining the mechanical properties and industrial processability of PLA-based composites.^24^ In addition, the thermal behavior of the neat GUR grades was examined to confirm their melt state during compounding. As shown in Figure 5, GUR 4120 and GUR 2126–2 exhibited a single melting peak with a melting temperature (*T*_m_), *T*_m_ = 133.0 and 134.6 °C, respectively. As the compounding temperature used in this work (145 °C) exceeds *T*_m_ for both grades, it can be stated that the GUR particles were in molten state during blends preparation. Nevertheless, UHMWPE have been in general shown to exhibit extremely high melt viscosity. Consequently, molten UHMPWE particles or fibers have been observed to retain their shape rather than to exhibited significant flow during their blending with another material, as postulated in the literature.^14^

**Figure 5:**
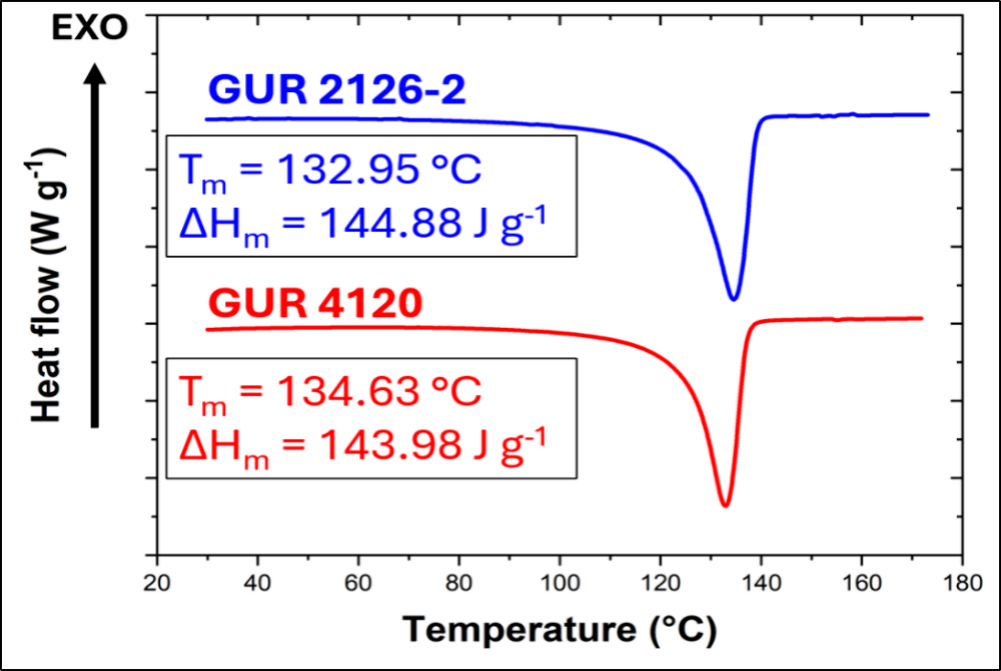
Second-heating DSC curves for neat GUR 4120 and GUR 2126–2 measured at a heating rate of 10 °C min^–1^; *T*_m_ and melting enthalpy (Δ*H*_m_) are indicated.

Figure 6 presents the DSC curves for the PLA_O and PLA/GUR samples. While the neat PLA_O exhibited no discernible crystallization during cooling at the applied cooling rate (20 °C min^–1^), all PLA/GUR blends demonstrated clear crystallization, with the crystallization temperature (*T*_c_) and crystallization enthalpy (Δ*H*_c_) increasing with GUR loading (see Supporting Information - Table S1–S4). This behavior suggests that the GUR particles acted as heterogeneous nucleation sites for PLA, which is consistent with previous reports that fillers can accelerate PLA crystallization further enhancing cell stabilization in the polymer foaming process, as demonstrated by Liu et al.^25^

**Figure 6:**
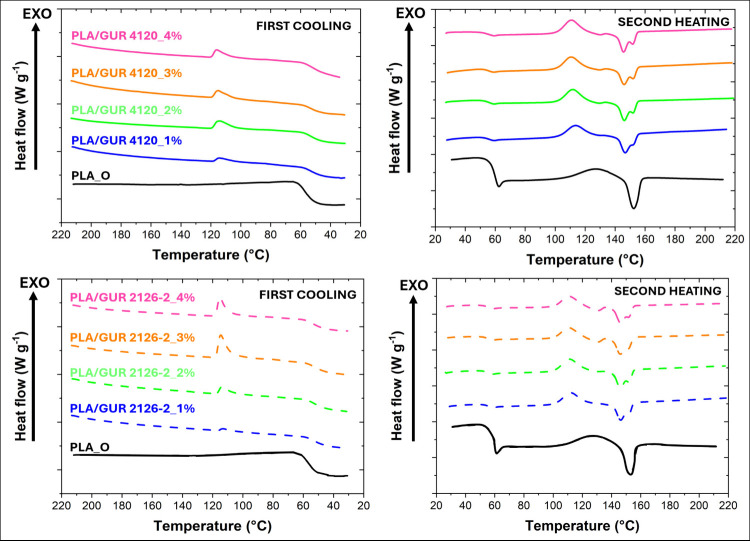
First-cooling and second-heating DSC curves of PLA_O and PLA/GUR (1–4 wt %) measured at a cooling rate of 20 °C min^–1^ and a heating rate of 10 °C min^–1^.

Two significant aspects were particularly pronounced upon heating when compared the PLA_O with the PLA/GUR samples. Initially, an endothermic peak associated with GUR melting is observed at approximately 130–135 °C, and its magnitude is found to scale with the GUR content. Second, the melting profile of PLA differed among the samples. Neat PLA_O exhibited a single melting peak, while PLA/GUR blends showed a double melting peak. The latter is possibly a well-known phenomenon in the PLA that arises from the coexistence of two crystalline phases, α-orthorhombic and α’-pseudo-orthorhombic, which exhibit different thicknesses and degrees of perfection.^26^ The presence of GUR has been demonstrated to induce such dual morphological states in PLA, which aligns with established interpretations of PLA double-peak melting behavior. Nevertheless, for the PLA/GUR composites, the crystallization enthalpy (during the cooling process) as well as the cold crystallization enthalpy (during subsequent heating) increased with higher GUR content. However, the observed melting enthalpy of PLA was found to be lower than the overall sum of the crystallization enthalpy, primarily at concentrations of GUR particles of ≥ 5 wt %. This apparent imbalance arises because PLA and GUR crystallization and melting occurred in similar (overlapping) temperature ranges. Thus, the exothermic process during the cooling of the materials sums the crystallization of both phases. Furthermore, the cold crystallization of PLA/GUR composites is influenced by GUR melting; for details, see Figure S6. However, even an addition of only 1% of GUR into PLA led to an increase in cold crystallization connected with increase in melting enthalpy from 5.73 J g^–1^ to 27.96 J g^–1^ for PLA_O and PLA/GUR 4120_1%, respectively. There is thus no doubt that the GUR particles induced the PLA crystallization process. The exclusion of PLA_R material is deliberate, serving to accentuate the contrast between the virgin PLA_O and the PLA/GUR samples. For the sake of clarity, quantitative values are comprehensively outlined in tables located in the Supporting Information.

### 3.4 Rheological Behavior Evaluated Using Rotational Rheometry

The viscoelastic behavior of the blends, incorporating both untreated and hydrophilic surface-treated particles, was characterized alongside original PLA (PLA_O) and reprocessed PLA (PLA_R). The rheological properties of these blends are of critical importance, as they directly impact material processability and foamability.

As illustrated in Figure 7, there is a clear trend of increasing complex viscosity with the presence of GUR particles. A higher filler concentration resulted in greater viscosity, attributed to restricted polymer chain mobility.^27^ An increase in matrix viscosity can be beneficial for PLA foam stability, as it prevents premature bubble coalescence and collapse during foaming. It is also important to note a slight decrease in complex viscosity for the PLA_R sample compared to PLA_O. This reduction, attributed to degradation during processing, is consistent with observations made in the previous section on blend morphology.

**Figure 7:**
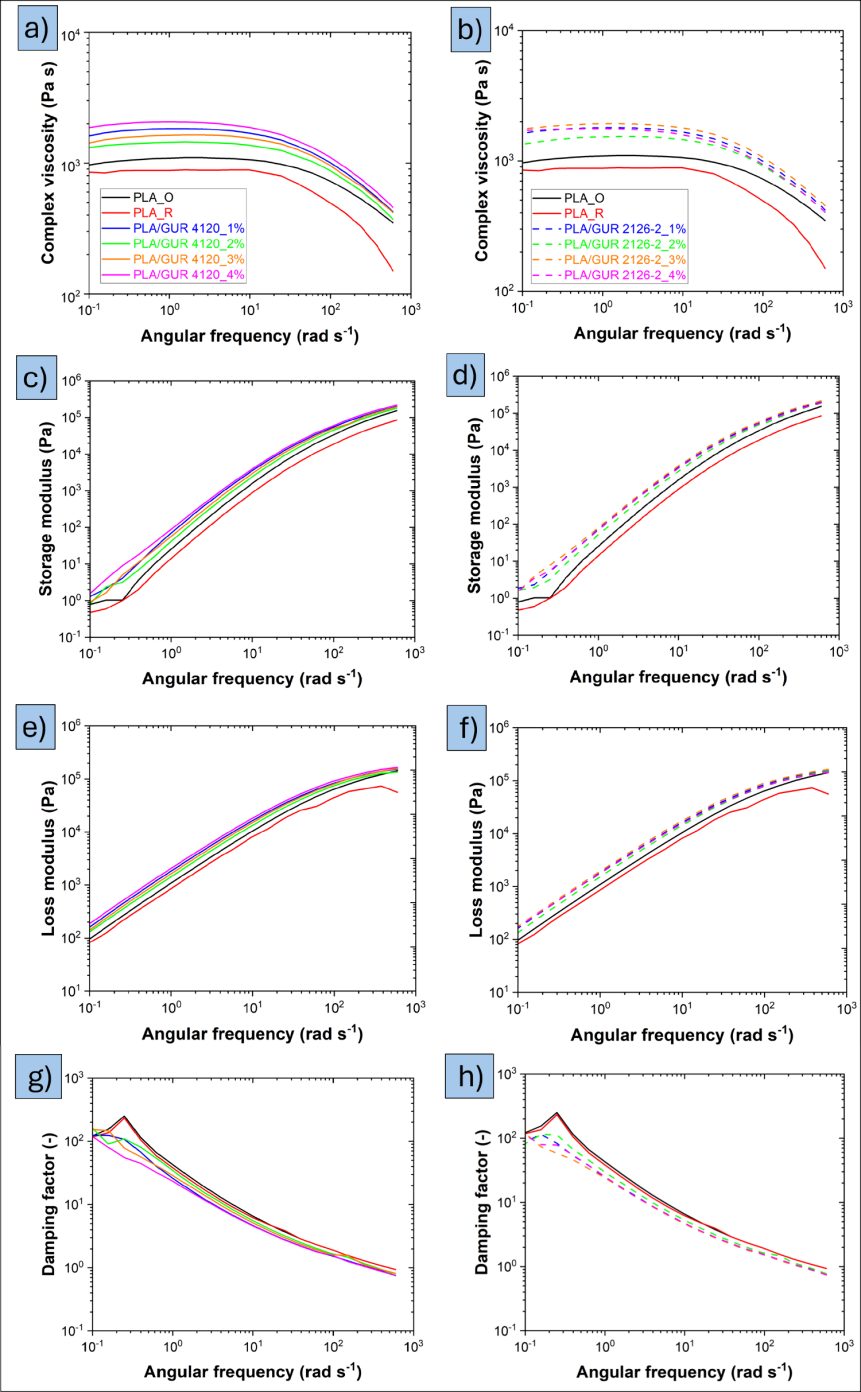
Rheological properties of PLA and PLA/GUR composites at 200 °C: (a, b) Complex viscosity for PLA/GUR 4120 and PLA/GUR 2126–2; (c, d) Storage modulus for PLA/GUR 4120 and PLA/GUR 2126–2; (e, f) Loss modulus for PLA/GUR 4120 and PLA/GUR 2126–2, (g, h) Damping factor for PLA/GUR 4120 and PLA/GUR 2126–2, at four representative loadings.

Regarding the storage modulus (*G’*), an increase indicates greater melt elasticity, which benefits foam processing by reducing cell-wall rupture and coalescence and stabilizing the cellular structure during foaming. A moderate increase in the loss modulus (*G’’*) can also be beneficial because it facilitates bubble nucleation in the initial stage of foaming.^28^ Overall, finding the right balance - sufficiently high *G′* for stability and not excessively large *G″* to ensure stable growth - is a key for proper foamability of the system. Damping factor (tan δ = *G″*/*G′*) was lower for all PLA/GUR blends than for neat PLA (Figure 7g, h), evidencing a shift toward a more elastic, energy-storing melt response. Reduced viscous dissipation (lower tan δ) is beneficial for foaming, as it stabilizes cell walls and suppresses coalescence during growth. The minimum values are observed for the 3–4 wt % samples, consistent with stronger morphology-driven reinforcement at the higher filler contents.^29,30^ This rheological window underpins the observed improvements in foamability upon adding small amounts of GUR.

### 3.5 Extensional Viscosity of PLA and PLA/GUR Composites Evaluated Using the Sentmanat Rheometer

Extensional tests were performed at 200 °C, a temperature well above the melting point of the PLA matrix and GUR particles. This ensured testing in the true melt regime for both phases. Experiments at substantially lower set temperatures did not provide reliable mounting/softening of the sample on the rotating SER drums (incomplete contact and slippage). Therefore, 200 °C was selected as a compromise for reproducible extensional measurements. Although neat PLA exhibits limited melt strength at 200 °C, the tendency to sag was kept minimal by a short preheat time before rotation (20 s – exact time control for each sample). It should be noted that to verify the reproducibility of the measured results, each of the experiments was performed at least 3 times, and after obtaining ≥ 3 consistent transient curves, we proceeded to the next experiment. Runs were programmed for two full drum revolutions to ensure a consistent end-condition (complete rupture). In line with SER best practice and specifications, the transient extensional viscosity reported here is evaluated within the first full revolution.^31^

Figure 8 shows extensional viscosity at 200 °C versus UHMWPE content exhibiting the highest values at 2 wt % and declining at higher loadings. Notably, samples at lower filler concentrations exhibited approximately a 3-fold increase in extensional viscosity on average, compared to PLA_O. It is important to note that no extensional data could be obtained for the reprocessed PLA_R, which repeatedly failed before reaching the target Hencky strains, which is consistent with reprocessing-induced molecular-weight reduction and the associated loss of melt strength reported for PLA. In contrast, adding just 1 wt % of GUR particles restored measurable extensional response and significantly enhanced the melt strength, with a maximum increase of 5.3-fold for the sample of PLA/GUR 2126–2_2%.

**Figure 8:**
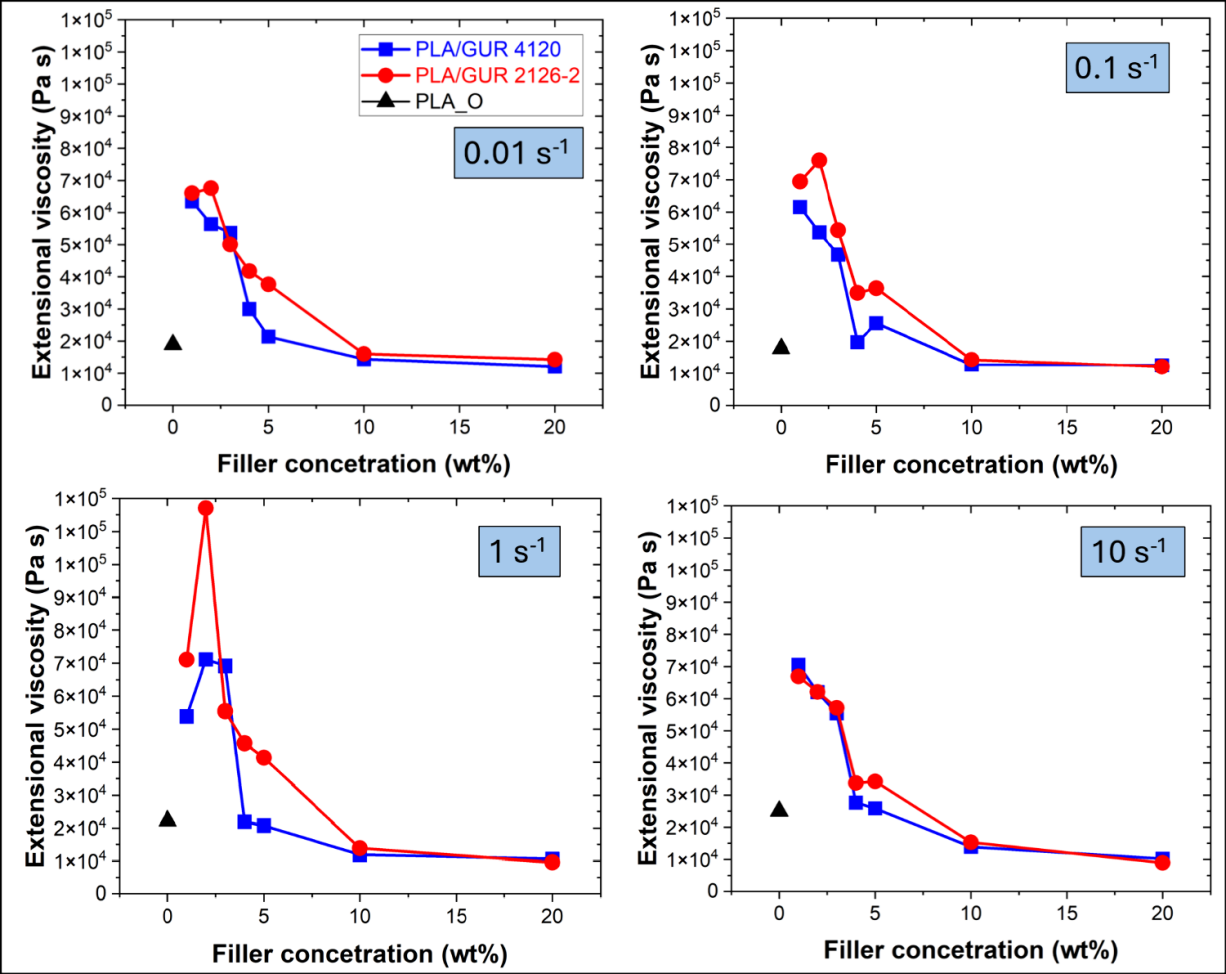
Extensional viscosity of PLA and PLA/GUR composites at 200 °C over Hencky strain rates (0.01–10 s^–1^), plotted versus UHMWPE content. PLA_R is not shown because extensional viscosity could not be measured reliably (insufficient melt strength).

Across most filler concentrations, surface-treated GUR 2126–2 samples exhibit higher extensional viscosity than untreated GUR 4120, likely due to finer particle size and improved interfacial adhesion/compatibility, which enhance stress transfer and suppress premature necking.^32^ These findings suggest that PLA blends containing surface-treated GUR particles may be more suitable for foam production, as they promote increased melt strength, a critical parameter in the foaming process.

### 3.6 Density of PLA and PLA/GUR Foams

Foam density strongly depends on polymer–filler interactions, foaming conditions, and the melt rheology of the matrix, and is further a critical parameter influencing thermal insulation and overall structural performance.^33^ Foam density was determined by hydrostatic weighing on a Radwag XA 52 balance equipped with a density kit, recording the mass in air and in deionized water at 21 ± 0.5 °C.

In Figure 9, the densities of PLA/GUR samples are presented. It can be seen that the lowest density was achieved at the UHMWPE concentration of 1 wt %. In general, higher filler loadings are associated with higher density, in accordance with prior reports.^34^ Compared with pure PLA foams, low GUR contents can reduce density, likely via enhanced bubble nucleation and expansion. Lower-density foams typically offer improved thermal insulation and energy absorption, and reduce material usage, contributing to sustainability in polymer engineering.^35^

**Figure 9:**
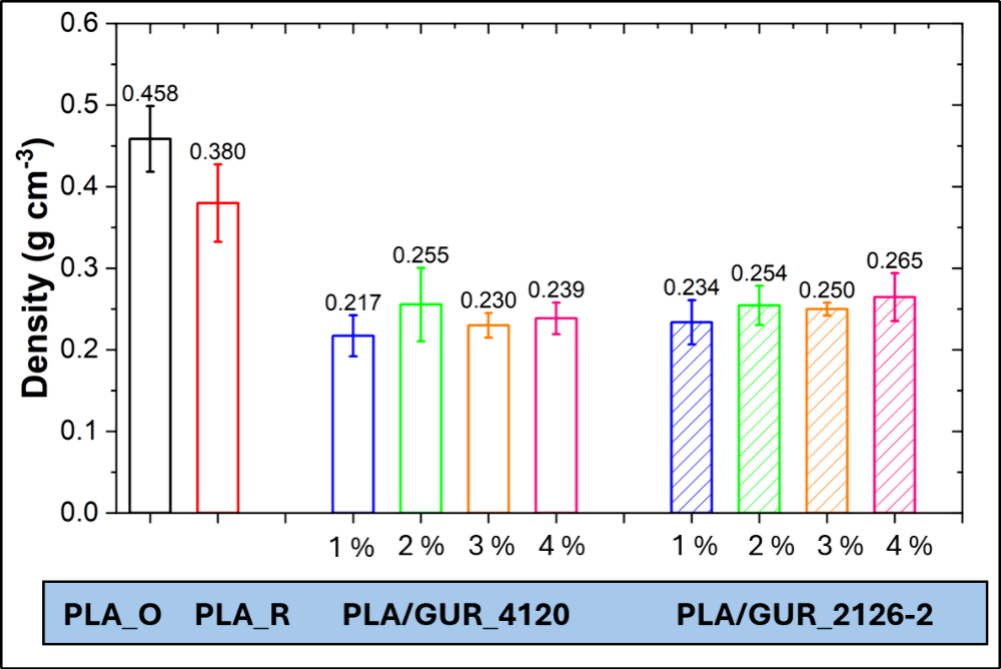
Density of PLA and PLA/GUR foams containing untreated GUR 4120 particles and hydrophilically surface-treated GUR 2126–2 particles at only four representative loadings for data clarity.

Although PLA_R reached a lower apparent density than PLA_O, the thermal insulation properties of polymer foams are governed by both density and cell morphology. Consequently, a lower density does not necessarily lead to lower thermal conductivity or a superior foam. Reprocessing of PLA_R reduced melt strength, which promotes cell-wall rupture and coalescence, producing polygonal, open cells. This morphology degrades both the mechanical integrity and thermal performance of the prepared foam, despite the lower density observed for PLA_R.

### 3.7 Thermal Conductivity of PLA and PLA/GUR Foams

The thermal conductivity of polymer foams is a key feature influencing their suitability for insulation and packaging applications. This property is primarily affected by foam density and pore morphology, both of which play a crucial role in determining heat transfer mechanisms within the material.^36^ As can be seen in Figure 10, a substantial reduction in thermal conductivity was observed in foams with the lowest GUR particle concentration. At 1 wt %, the untreated GUR 4120 sample reached 0.019 W m^–1^ K^–1^, whereas the surface-treated GUR 2126–2 showed 0.021 W m^–1^ K^–1^. Only four representative loadings with low thermal conductivity are plotted for clarity; above 5 wt %, the thermal conductivity starts to rise. Notably, the difference between the references is modest (0.033 W m^–1^ K^–1^ for PLA_O vs 0.030 W m^–1^ K^–1^ for PLA_R), which is in sharp contrast to the findings observed in the density results. This finding suggests that, despite the reprocessing reduced density of PLA_R foams, the more irregular and open-cell structure of PLA_R decreased insulation properties.

**Figure 10:**
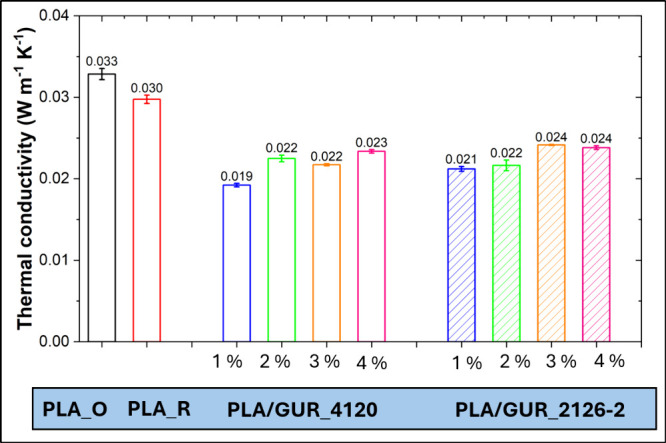
Thermal conductivity of PLA and PLA/GUR foams containing untreated GUR 4120 particles and hydrophilically surface-treated GUR 2126–2 particles (four loadings are shown for clarity).

In comparison to pure PLA, a significant reduction in thermal conductivity was observed in PLA/GUR foams with lower GUR concentrations, indicating an enhancement in thermal insulation performance. This decrease is attributed to a lower solid-phase conduction connected with the reduced density and, more importantly, to a finer, more regular, predominantly closed-cell morphology promoted by particle-assisted nucleation and higher melt strength. By contrast, higher UHMWPE loadings (≥5 wt %) increased thermal conductivity because the solid conduction pathway grows with increasing foam density, and agglomeration can coarsen cells, increasing radiative transport.^37^

### 3.8 Impact Strength of PLA and PLA/GUR Foams

The impact strength of polymer foams is a critical parameter that determines their capacity to withstand sudden or dynamic loads, rendering it a crucial property for applications in the automotive and packaging industries.^38^ It is a well-established fact that higher foam density is generally associated with increased impact resistance, thereby providing a more significant solid fraction per unit volume to absorb impact energy. Nevertheless, polymer foams with well-structured pore morphology have been demonstrated to enhance energy dissipation, thereby improving the mechanical resistance of the foam.^39^ For instance, in a study by Bao et al.,^40^ it was proposed that a bimodal cell structure could exhibit superior performance in comparison to uniform-cell foams when the proportion of large pores falls within the range of 25–32% and their diameter remains below 25 μm.

Figure 11 shows the dependence of the PLA/GUR foams impact strength on the UHMWPE concentration. It is evident that at low UHMWPE concentrations, the prepared foams exhibited impact strength comparable with original PLA, or even exceeding it in some cases. However, with further increases in filler content (>2–3%) the impact strength of the prepared foams significantly decreased.

**Figure 11:**
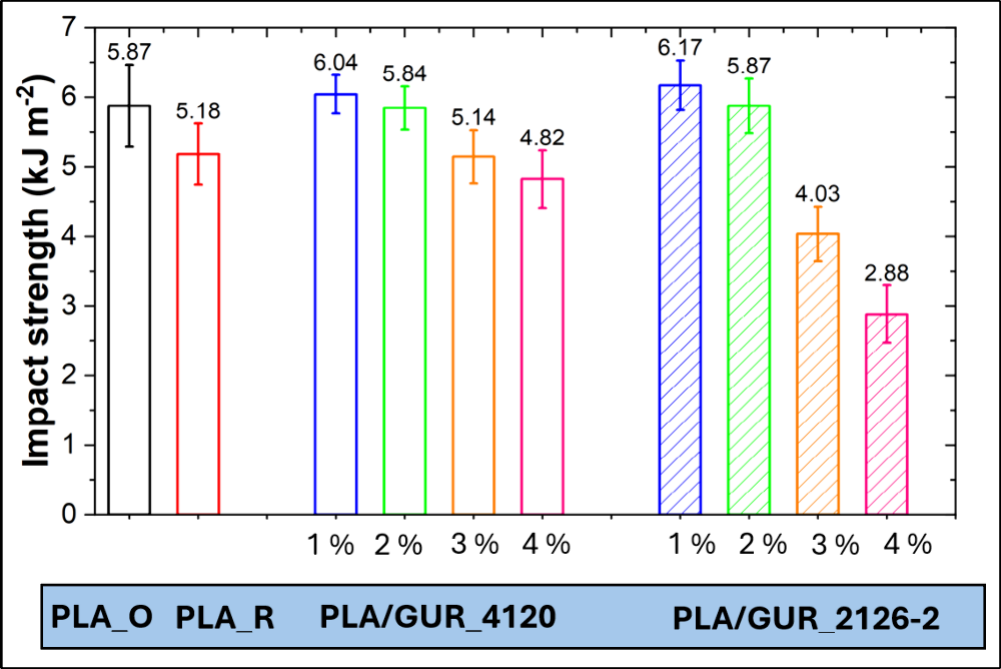
Impact strength of PLA and PLA/GUR foams containing untreated GUR 4120 particles and hydrophilically surface-treated GUR 2126–2 particles at selected concentrations.

The highest impact strength was obtained for the PLA/GUR 2126–2_1% sample with a more compatible surface-treated UHMWPE, which combines a bimodal cell morphology. This unique configuration likely resulted in a better balance of energy absorption and dissipation, producing a foam with higher impact resistance (6.17 kJ m^–2^) compared to pure PLA foam (5.87 kJ m^–2^). By contrast, PLA_R (reprocessed PLA) showed lower impact strength, consistent with its irregular/polygonal and more open cells.

Based on the aforementioned findings, the prepared foams with low GUR concentrations exhibited higher or comparable impact strength, lower apparent density and improved thermal insulation when compared with the foam prepared from original PLA. Interestingly, the large-cell fraction reached approximately 43% at low GUR contents, with an average large-cell diameter of 100 μm, which exceeds the window suggested by Bao et al.^40^ for superior foams. This thus indicates a scope for further optimization concerning a reduction of the large cell sizes, which should further increase the impact strength.

## 4 Conclusion

This work demonstrates a simple polymer–polymer composite strategy to restore and enhance the melt strength of recycled PLA by incorporating small amounts of UHMWPE. Even an addition of only 1 wt % UHMWPE enabled measurement of the extensional response of reprocessed PLA, which was otherwise unmeasurable due to degradation during reprocessing. Melt strength was fully recovered and further increased by up to 5.3-fold, enabling stable cell growth and expansion during foaming. Furthermore, an effect of UHMWPE particle concentration, particle size and their surface treatment on the melt strength and foamability of the prepared polymer–polymer composites has been investigated. The addition of UHMWPE generally resulted in the formation of a bimodal porous structure, where systems with a fraction of large pores below 43% exhibited, after reprocessing, impact strength comparable with or higher than that of the original PLA. As a result, the prepared foams exhibited a thermal conductivity as low as 0.019 W m^–1^ K^–1^ and a density of 0.217 g cm^–3^, while maintaining or exceeding impact strength comparable to the original PLA foams.

In conclusion, it is evident that the utilization of PLA/UHMWPE composites facilitates a scalable and industry-compatible approach to convert recycled PLA into lightweight, thermally insulating foams. These foams find application in protective packaging, with the advantage of reduced material usage due to their lower density. In future research, further refinement of the large-cell population (size and fraction) is anticipated to yield substantial enhancement of mechanical performance without compromising thermal insulation efficiency.

## Supplementary Material



## Notes

Additional figures and data for all GUR loadings (0–20 wt %): Figure S1, areal fraction of large pores vs GUR loading; Figure S2. DSC first-cooling scans for PLA_O and PLA/GUR 4120; Figure S 3. DSC first-cooling scans for PLA_O and PLA/GUR 2126–2; Figure S 4. DSC second-heating scans for PLA_O and PLA/GUR 4120; Figure S 5. DSC second-heating scans for PLA_O and PLA/GUR 2126–2; Table S 1–4. Specific DSC values; Figure S6, DSC curves for neat GUR 4120, neat PLA 2003, and PLA/GUR 4120 _20 wt %; Figure S7–S8, complex viscosity vs angular frequency at 200 °C for PLA/GUR 4120 and PLA/GUR 2126–2; Figure S9–S10, storage modulus vs angular frequency at 200 °C for PLA/GUR 4120 and PLA/GUR 2126–2; Figure S11–S12, loss modulus vs angular frequency at 200 °C for PLA/GUR 4120 and PLA/GUR 2126–2; Figure S13–S14, damping factor vs angular frequency at 200 °C for PLA/GUR 4120 and PLA/GUR 2126–2; Figure S15, foam density vs GUR loading; Figure S16, thermal conductivity vs GUR loading; Figure S17, impact strength vs GUR loading; Figure S18, Schematic of the batch foaming apparatus; Figure S19, Batch foaming setup; Figure S20, Schematic of the batch foaming chamber (PDF)

## References

[1] Taib N.-A. A. B., Rahman M. R., Huda D., Kuok K. K., Hamdan S., Bakri M. K. B., Julaihi M. R. M. B., Khan A. (2023). A review on poly lactic
acid (PLA) as a biodegradable polymer. Polym.
Bull..

[2] Swetha T. A., Bora A., Mohanrasu K., Balaji P., Raja R., Ponnuchamy K., Muthusamy G., Arun A. (2023). A comprehensive review
on polylactic acid (PLA) - Synthesis, processing and application in
food packaging. Int. J. Biol. Macromol..

[3] Romani A., Perusin L., Ciurnelli M., Levi M. (2024). Characterization of
PLA feedstock after multiple recycling processes for large-format
material extrusion additive manufacturing. Materials
Today Sustainability.

[4] Aguero A., Morcillo M. d. C., Quiles-Carrillo L., Balart R., Boronat T., Lascano D., Torres-Giner S., Fenollar O. (2019). Study of the Influence
of the Reprocessing Cycles on the Final Properties of Polylactide
Pieces Obtained by Injection Molding. Polymers.

[5] Yao X., Yang X., Lu Y., Qiu Y., Zeng Q. (2025). Review of
the Synthesis and Degradation Mechanisms of Some Biodegradable Polymers
in Natural Environments. Polymers.

[6] Wu F., Misra M., Mohanty A. K. (2021). Challenges
and new opportunities
on barrier performance of biodegradable polymers for sustainable packaging. Prog. Polym. Sci..

[7] Wang G., Zhao J., Wang G., Zhao H., Lin J., Zhao G., Park C. B. (2020). Strong and super thermally insulating
in-situ nanofibrillar PLA/PET composite foam fabricated by high-pressure
microcellular injection molding. Chemical engineering
journal (Lausanne, Switzerland: 1996).

[8] Liu W., Wu X., Zhu J., Chen X., Liu S., Li Y. (2024). Enhancing
foamability and flame-retardancy of polylactic acid bead foams through
inter-beads bulk polymerization and continuous phase dispersion of
LDHs. Journal of polymer science (2020).

[9] Mousavi S. M., Ahmadi S., Rasouli S., Fasihi M. (2024). Improving foamability
and foam stability of poly­(ethylene terephthalate) through chemical
modification with styrene maleic anhydride. J. Appl. Polym. Sci..

[10] Wang Z., Wang G., Xu Z., Chai J., Zhao G. (2025). Innovative
crosslinking and foaming Strategies for Advancing biodegradable composite
foams: Enhancing Foamability, Flexibility, and thermal insulation. Materials & design.

[11] Azdast T., Hasanzadeh R. (2021). Increasing cell density/decreasing cell size to produce
microcellular and nanocellular thermoplastic foams: A review. Journal of Cellular Plastics.

[12] Hou J., Zhao G., Wang G. (2021). Polypropylene/talc
foams with high
weight-reduction and improved surface quality fabricated by mold-opening
microcellular injection molding. Journal of
materials research and technology.

[13] Chand N., Naik A. M., Khaira H. K. (2007). Development of UHMWPE
modified PP/PET
blends and their mechanical and abrasive wear behavior. Polym. Compos..

[14] Amza C. G., Zapciu A., Eypórsdóttir A., Björnsdóttir A., Borg J. (2019). Embedding Ultra-High-Molecular-Weight Polyethylene Fibers in 3D-Printed
Polylactic Acid (PLA) Parts. Polymers.

[15] Malkin A. Y., Ladygina T. A., Gusarov S. S., Dudka D. V., Mityukov A. V. (2024). Characterization
and Rheological Properties of Ultra-High Molecular Weight Polyethylenes. Polymers.

[16] Richards E., Rizvi R., Chow A., Naguib H. (2008). Biodegradable Composite
Foams of PLA and PHBV Using Subcritical CO2. J. Polym. Environ.

[17] Turicek J., Ratts N., Kaltchev M., Masoud N. (2021). Surface Treatment
of
Ultra-High Molecular Weight Polyethylene (UHMWPE) by Cold Atmospheric
Plasma (CAP) for Biocompatibility Enhancement. Applied sciences.

[18] Wu M., Ren Q., Zhu X., Li W., Luo H., Wu F., Wang L., Zheng W., Cui P., Yi X. (2023). Super toughened
blends of poly­(lactic acid) and poly­(butylene adipate-co-terephthalate)
injection-molded foams via enhancing interfacial compatibility and
cellular structure. Int. J. Biol. Macromol..

[19] Zhao J., Wang G., Chen Z., Huang Y., Wang C., Zhang A., Park C. B. (2021). Microcellular
injection molded outstanding
oleophilic and sound-insulating PP/PTFE nanocomposite foam. Composites. Part B, Engineering.

[20] Cuadra-Rodríguez D., Barroso-Solares S., Rodríguez-Pérez M. A., Pinto J. (2022). Production of cellular
polymers without solid outer skins by gas
dissolution foaming: A long-sought step towards new applications. Materials & design.

[21] Porfiri M., Gupta N. (2009). Effect of volume fraction and wall thickness on the elastic properties
of hollow particle filled composites. Composites.
Part B, Engineering.

[22] Yeh S., Demewoz N. M., Kurniawan V. (2021). Controlling
the structure and density
of PMMA bimodal nanocellular foam by blending different molecular
weights. Polym. Test..

[23] Zhao J., Qiao Y., Wang G., Wang C., Park C. B. (2020). Lightweight
and tough PP/talc composite foam with bimodal nanoporous structure
achieved by microcellular injection molding. Materials & design.

[24] Wang G., Zhang D., Li B., Wan G., Zhao G., Zhang A. (2019). Strong and thermal-resistance glass fiber-reinforced polylactic acid
(PLA) composites enabled by heat treatment. Int. J. Biol. Macromol..

[25] Liu W., He S., Yang Y. (2019). Effect of
stereocomplex crystal on foaming behavior
and sintering of poly­(lactic acid) bead foams. Polymer international.

[26] Mayouf I., Guessoum M., Rahem Z., Fuensanta M., Martin-Martinez J. M. (2022). Structural, thermo-mechanical and morphological properties
of composites made with poly­(lactic acid) and poly­(ethylene terephthalate)
fibers without compatibilizer. J. Adhes. Sci.
Technol..

[27] Perera Y. S., Naaib M., Ariyasinghe N., Abeykoon C. (2025). Investigation of the
effect of extrusion process parameters and filler loading on the performance
of LDPE composites reinforced with eggshell powder. Composites. Part C, Open access.

[28] Feng J. J., Bertelo C. A. (2004). Prediction of bubble growth and size
distribution in
polymer foaming based on a new heterogeneous nucleation model. J. Rheol..

[29] Tian H., Wang Z., Yu J., Zhao Y., Pan H., Bian J., Yang H., Wang Z., Zhang H. (2025). Preparation
of high elastic bimodal cells biodegradable foam. Polymer (Guilford).

[30] Chai J., Wang G., Wei C., Li X., Shao R., Zhao G. (2025). Ultra-expanded microcellular shape memory polymer foams via supercritical
foaming for recyclable oil absorption and improved thermal insulation. Advanced industrial and engineering polymer research.

[31] Küchenmeister-Lehrheuer, C. ; Meyer, F. Sentmanat Extensional Rheometer (SER) for HAAKE MARS Rheometers; Product Information P019; Thermo Fisher Scientific, 2023. Available at: https://documents.thermofisher.com/TFS-Assets/MSD/Specification-Sheets/P019-sentmanat-extensional-rheometer-ser-haake-mars-rheometer.pdf (accessed 2025–10–31).

[32] Munstedt H. (2024). Melt strain
hardening of polymeric systems filled with solid particles: review
and supplementary experimental results. Rheol.
Acta.

[33] Valipour F., Dehghan S. F., Hajizadeh R. (2022). The effect of nano- and microfillers
on thermal properties of Polyurethane foam. Int. J. Environ. Sci. Technol..

[34] Zhang H., Zhang G., Tang M., Zhou L., Li J., Fan X., Shi X., Qin J. (2018). Synergistic effect
of carbon nanotube
and graphene nanoplates on the mechanical, electrical and electromagnetic
interference shielding properties of polymer composites and polymer
composite foams. Chemical Engineering Journal.

[35] Chaib M., Thakur S., Youcef H. B., Lahcini M., Verdejo R. (2025). Achieving
rapid foaming in self-blown non-isocyanate polyurethane foams via
controlled epoxy functionality in cyclic carbonates. European polymer journal.

[36] Demori R., Bischoff E., de Azeredo A. P., Liberman S. A., Maia J., Mauler R. S. (2018). Morphological, thermo-mechanical,
and thermal conductivity
properties of halloysite nanotube-filled polypropylene nanocomposite
foam. Journal of cellular plastics.

[37] Santiago-Calvo M., Tirado-Mediavilla J., Rauhe J. C., Jensen L. R., Ruiz-Herrero J. L., Villafañe F., Rodríguez-Pérez MÁ. (2018). Evaluation
of the thermal conductivity and mechanical properties of water blown
polyurethane rigid foams reinforced with carbon nanofibers. European polymer journal.

[38] Chen Y., Wu L. (2025). High Glass Transition
PEcBT and PEPecBT Copolyesters Synthesized
by Direct Esterification Route. J. Appl. Polym.
Sci..

[39] Wang G., Zhang D., Wan G., Li B., Zhao G. (2019). Glass fiber
reinforced PLA composite with enhanced mechanical properties, thermal
behavior, and foaming ability. Polymer (Guilford).

[40] Bao J., Weng G., Zhao L., Liu Z., Chen Z. (2014). Tensile and
impact behavior of polystyrene microcellular foams with bi-modal cell
morphology. Journal of cellular plastics.

