# Supplementary material for: UHMWPE-Assisted Melt Strength Enhancement of Recycled PLA for Lightweight Foaming
Source: ACS Polym Au. 2025 Nov 14;6(1):376–87. doi: 10.1021/acspolymersau.5c00154 (PMC12903422; doi:10.1021/acspolymersau.5c00154)
Supplement: Supplementary file 1 [file lg5c00154_si_001.pdf]

Supporting information

UHMWPE-assisted melt strength enhancement of recycled PLA for lightweight foaming

Rostislav Vilem[a*], Ondrej Mertlik[a], Tomas Plachy[a], Lukas Manas[a], Tomas Sedlacek[a]

[a] Centre of Polymer Systems of Tomas Bata University in Zlín, Tř. T. Bati 5678, Zlín 760 01, Czech Republic

r_vilem@utb.cz (R.V.); o_mertlik@utb.cz (O.M.); plachy@utb.cz (T.P.); lmanas@utb.cz (L.M.) sedlacek@utb.cz (T.S.)

*Corresponding author: r_vilem@utb.cz (R.V.)

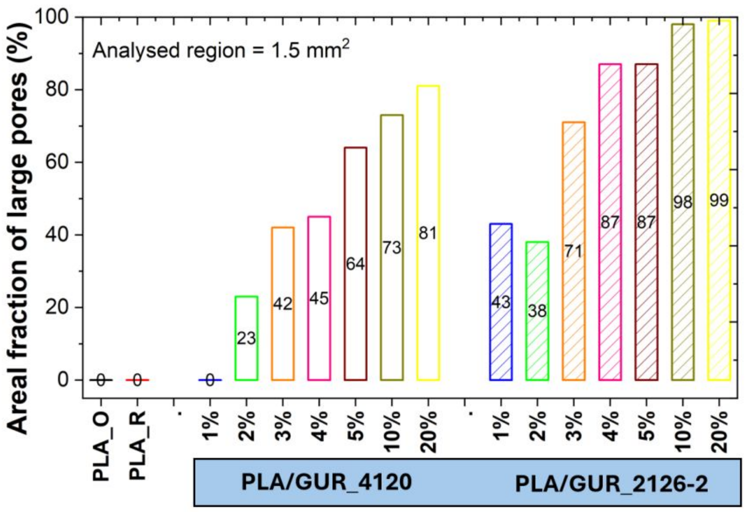

Fig. S 1. Comparison of the areal fraction of large pores in PLA and PLA/GUR composite foams at filler loadings 0–20 wt%.

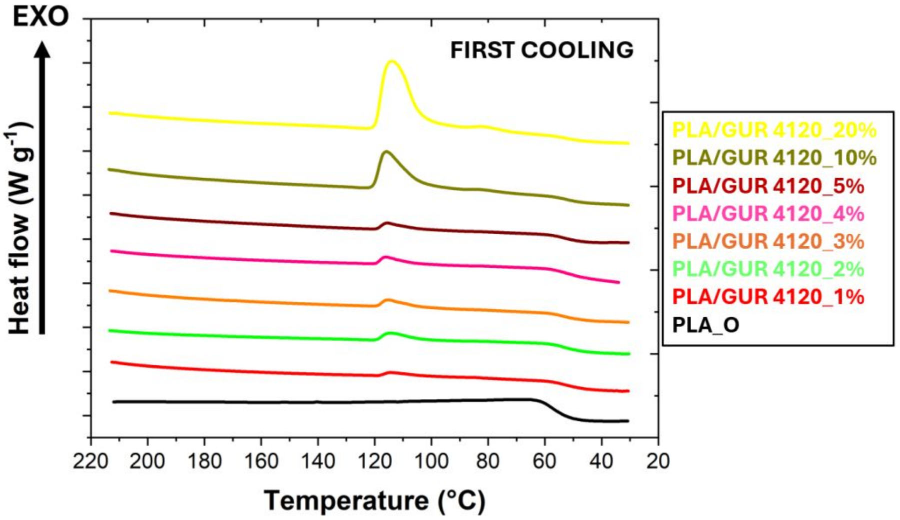

Fig. S 2. DSC first-cooling scans for PLA_O and PLA/GUR 4120 (1–20 wt%); 220 → 20 °C at 20 °C min⁻¹.

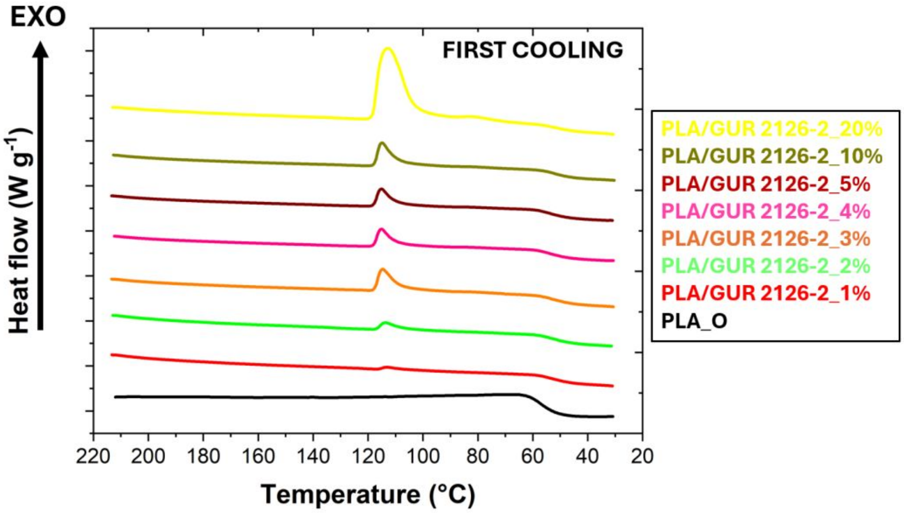

Fig. S 3. DSC first-cooling scans for PLA_O and PLA/GUR 2126-2 (1–20 wt%); 220 → 20 °C at 20 °C min⁻¹.

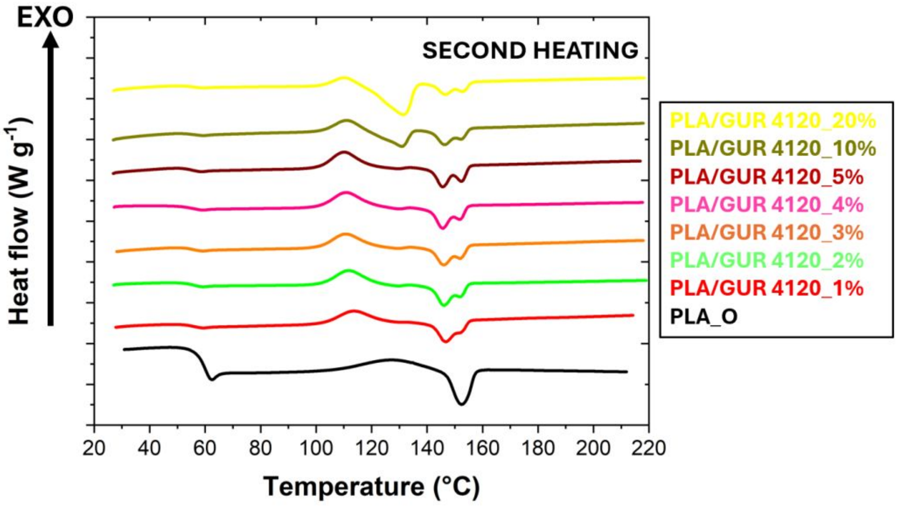

Fig. S 4. DSC second-heating scans for PLA_O and PLA/GUR 4120 (1–20 wt%); 20 → 220 °C at 10 °C min⁻¹.

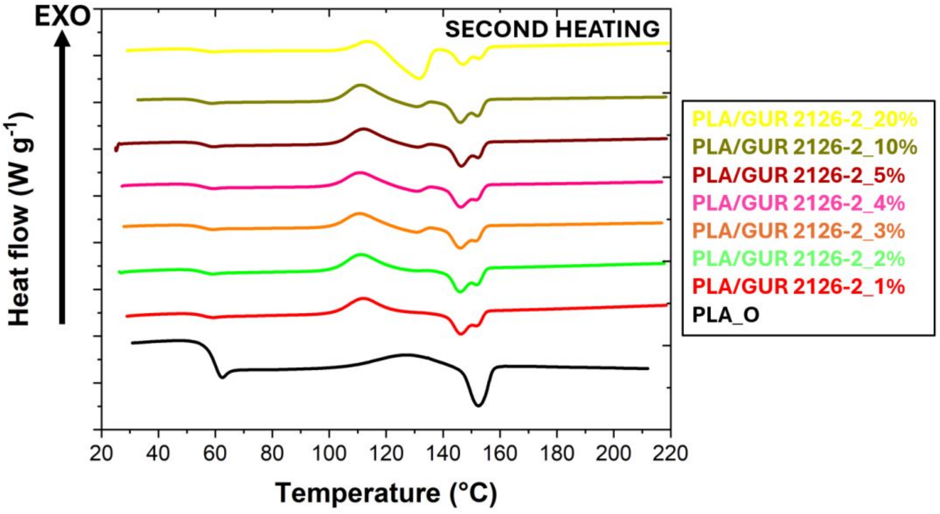

Fig. S 5. DSC second-heating scans for PLA_O and PLA/GUR 2126-2 (1–20 wt%); 20 → 220 °C at 10 °C min^{-1}.

Table S 1. Crystallization (T_c), melting (T_m), glass transition (T_g) and cold crystallization (T_{cc}) temperatures, crystallization enthalpy (ΔH_c), cold crystallization enthalpy (ΔH_{cc}) and melting enthalpy (ΔH_m) of PLA_O and PLA/GUR 4120 (1–4 wt%). It is important to note that the values mentioned in the tables below (**Tables S1–4**) are influenced by the overlapping temperature ranges of crystallization and melting of PLA and GUR. Thus, for example, the exothermic peak representing crystallization sums the crystallization of both phases. Furthermore, the cold crystallization of PLA/GUR composites is influenced by GUR melting and vice versa.

	**PLA_O**	**PLA/GUR 4120_1%**	**PLA/GUR 4120_2%**	**PLA/GUR 4120_3%**	**PLA/GUR 4120_4%**
**First cooling**					
**$T_{c, PLA}$ (°C)**	-	114.3	114.39	119.04	119.59
**$\Delta H_{c, PLA}$* (J/g)**	-	1.83	2.43	3.73	3.96
**Second heating**					
**T_g (°C)**	58.94	54.54	54.65	53.96	54.12
**$T_{cc, PLA}$ (°C)**	110.29	113.44	111.74	110.61	101.34
**$\Delta H_{cc, PLA}$ (J/g)**	4.85	25.22	26.88	27.34	28.05
**$T_{m1, PLA}$ (°C)**	152.46	146.81	146.23	146.15	145.60
**$T_{m2, PLA}$ (°C)**	-	150.86	151.23	151.33	151.47
**$\Delta H_{m, PLA}$ (J/g)**	5.73	27.96	27.65	25.84	26.27
**$T_{m, GUR}$ (°C)**	-	129.25	129.67	130.06	130.01
**$\Delta H_{m, GUR}$ (J/g)**	-	0.45	0.53	0.31	0.66

*ΔH_c represents the total crystallization enthalpy of the composite and includes contributions from both the PLA matrix and the GUR phase.

Table S 2. Crystallization (T_c), melting (T_m), glass transition (T_g) and cold crystallization (T_{cc}) temperatures, crystallization enthalpy (ΔH_c), cold crystallization enthalpy (ΔH_{cc}) and melting enthalpy (ΔH_m) of PLA/GUR 4120 (5–20 wt%) and GUR 4120.

	**PLA/GUR 4120_5%**	**PLA/GUR 4120_10%**	**PLA/GUR 4120_20%**	**GUR 4120**
**First cooling**				
$\mathbf{T_{c,\,PLA}}$ **(°C)**	115.24	115.72	114.20	120.32
$\mathbf{\Delta H_{c,\,PLA}}$*** (J/g)**	4.58	25.09	50.88	123.06
**Second heating**				
$\mathbf{T_g}$ **(°C)**	53.68	54.42	54.89	-
$\mathbf{T_{cc,\,PLA}}$ **(°C)**	109.95	110.94	109.92	-
$\mathbf{\Delta H_{cc,\,PLA}}$ **(J/g)**	27.85	17.89	10.05	-
$\mathbf{T_{m1,\,PLA}}$ **(°C)**	145.64	146.36	146.69	-
$\mathbf{T_{m2,\,PLA}}$ **(°C)**	152.20	152.08	152.22	-
$\mathbf{\Delta H_{m,\,PLA}}$ **(J/g)**	27.48	19.51	15.39	-
$\mathbf{T_{m,\,GUR}}$ **(°C)**	129.72	131.02	131.43	142.13
$\mathbf{\Delta H_{m,\,GUR}}$ **(J/g)**	0.55	17.51	47.73	126.75

*ΔH_c represents the total crystallization enthalpy of the composite and includes contributions from both the PLA matrix and the GUR phase.

Table S 3. Crystallization (T_c), melting (T_m), glass transition (T_g) and cold crystallization (T_{cc}) temperatures, crystallization enthalpy (ΔH_c), cold crystallization enthalpy (ΔH_{cc}) and melting enthalpy (ΔH_m) of PLA/GUR 2126-2 (1–4 wt%).

	**PLA/GUR 2126-2_1%**	**PLA/GUR 2126-2_2%**	**PLA/GUR 2126-2_3%**	**PLA/GUR 2126-2_4%**
**First cooling**				
$\mathbf{T_{c,\,PLA}}$ **(°C)**	112.94	113.68	114.43	114.95
$\mathbf{\Delta H_{c,\,PLA}}$ **(J/g)**	0.74	2.97	7.68	8.14
**Second heating**				
$\mathbf{T_g}$ **(°C)**	54.66	54.50	54.60	55.37
$\mathbf{T_{cc,\,PLA}}$ **(°C)**	111.77	111.08	110.72	111.22
$\mathbf{\Delta H_{cc,\,PLA}}$ **(J/g)**	27.83	27.47	24.22	25.14
$\mathbf{T_{m1,\,PLA}}$ **(°C)**	146.31	146.12	146.10	146.20
$\mathbf{T_{m2,\,PLA}}$ **(°C)**	150.36	141.02	151.69	151.66
$\mathbf{\Delta H_{m,\,PLA}}$ **(J/g)**	27.01	27.25	25.15	25.39
$\mathbf{T_{m,\,GUR}}$ **(°C)**	130.40	130.53	130.66	130.81
$\mathbf{\Delta H_{m,\,GUR}}$ **(J/g)**	0.16	0.33	3.36	1.17

*ΔH_c represents the total crystallization enthalpy of the composite and includes contributions from both the PLA matrix and the GUR phase.

Table S 4. Crystallization (T_c), melting (T_m), glass transition (T_g) and cold crystallization (T_{cc}) temperatures, crystallization enthalpy (ΔH_c), cold crystallization enthalpy (ΔH_{cc}) and melting enthalpy (ΔH_m) of PLA/GUR 2126-2 (5–20 wt%) and GUR 2126-2.

	**PLA/GUR 2126-2_5%**	**PLA/GUR 2126-2_10%**	**PLA/GUR 2126-2_20%**	**GUR 2126-2**
**First cooling**				
**$T_{c, PLA}$ (°C)**	115.10	114.78	112.92	118.36
**$\Delta H_{c, PLA*}$ (J/g)**	9.87	10.02	49.83	144.21
**Second heating**				
**T_g (°C)**	55.57	54.25	54.83	-
**$T_{cc, PLA}$ (°C)**	111.92	111.07	113.38	-
**$\Delta H_{cc, PLA}$ (J/g)**	25.44	25.93	11.29	-
**$T_{m1, PLA}$ (°C)**	146.40	146.08	146.97	-
**$T_{m2, PLA}$ (°C)**	151.96	150.49	152.83	-
**$\Delta H_{m, PLA}$ (J/g)**	25.59	25.64	17.92	-
**$T_{m, GUR}$ (°C)**	131.01	130.82	131.58	141.68
**$\Delta H_{m, GUR}$ (J/g)**	1.97	3.69	34.40	144.398

*ΔH_c represents the total crystallization enthalpy of the composite and includes contributions from both the PLA matrix and the GUR phase.

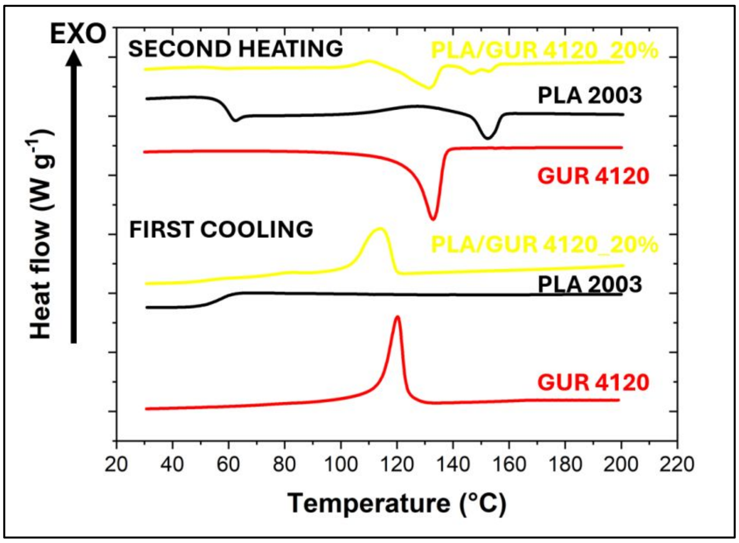

Fig. S 6. DSC curves from first cooling (20 °C min^{-1}) and second heating (10 °C min^{-1}) for neat GUR 4120, neat PLA 2003, and PLA/GUR 4120 _20 wt%. The data indicate that GUR crystallization and melting influence the crystallization and cold-crystallization peaks of the PLA/GUR composites.

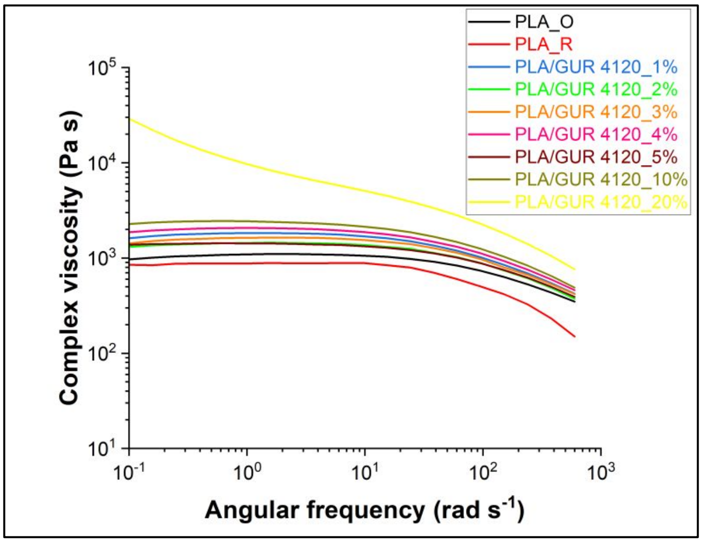

Fig. S 7. Rheological properties of PLA and PLA/GUR composites at GUR loading 0-20 wt% at 200 °C, a dependence of complex viscosity on the angular frequency for PLA/GUR 4120.

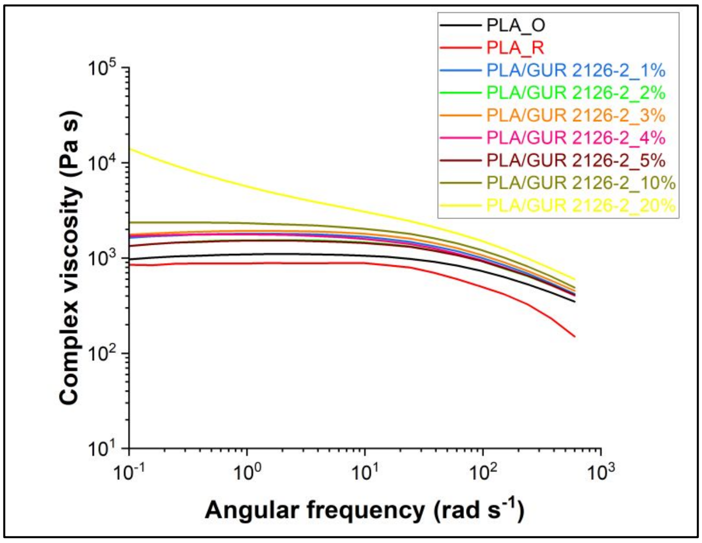

Fig. S 8. Rheological properties of PLA and PLA/GUR composites at GUR loading 0-20 wt% at 200 °C, a dependence of complex viscosity on the angular frequency for PLA/GUR 2126-2.

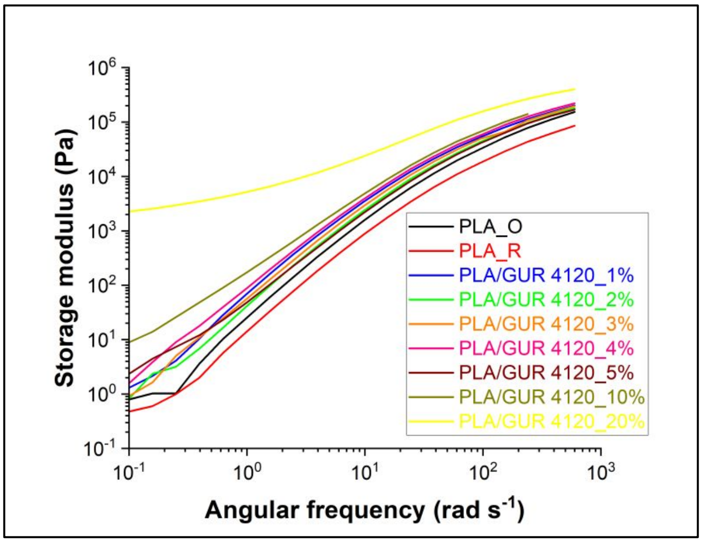

Fig. S 9. Rheological properties of PLA and PLA/GUR composites at GUR loading 0-20 wt% at 200 °C, a dependence of storage modulus on the angular frequency for PLA/GUR 4120.

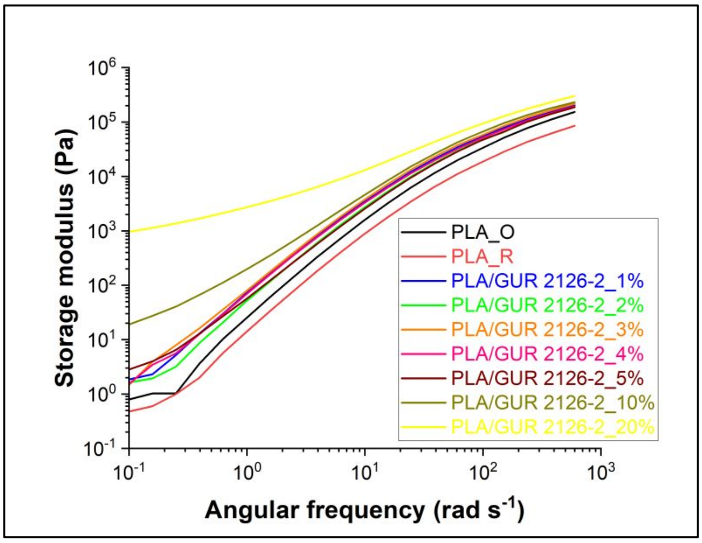

Fig. S 10. Rheological properties of PLA and PLA/GUR composites at GUR loading 0-20 wt% at 200 °C, a dependence of storage modulus on the angular frequency for PLA/GUR 2126-2.

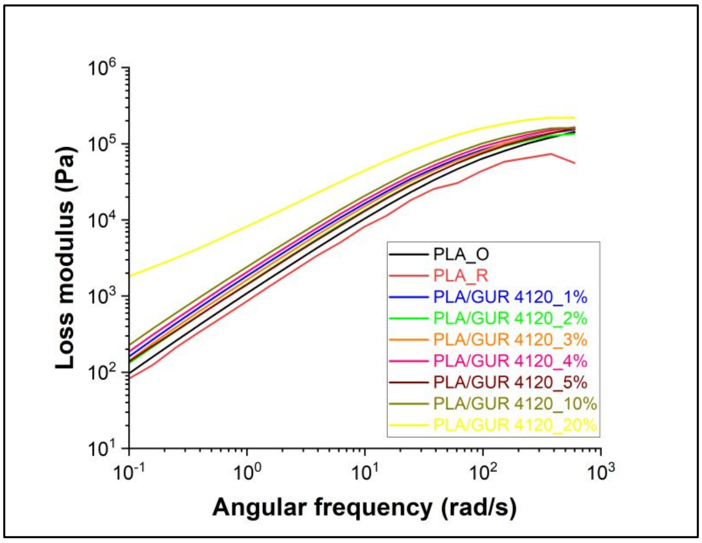

Fig. S 11. Rheological properties of PLA and PLA/GUR composites at GUR loading 0-20 wt% at 200 °C, a dependence of loss modulus on the angular frequency for 4120.

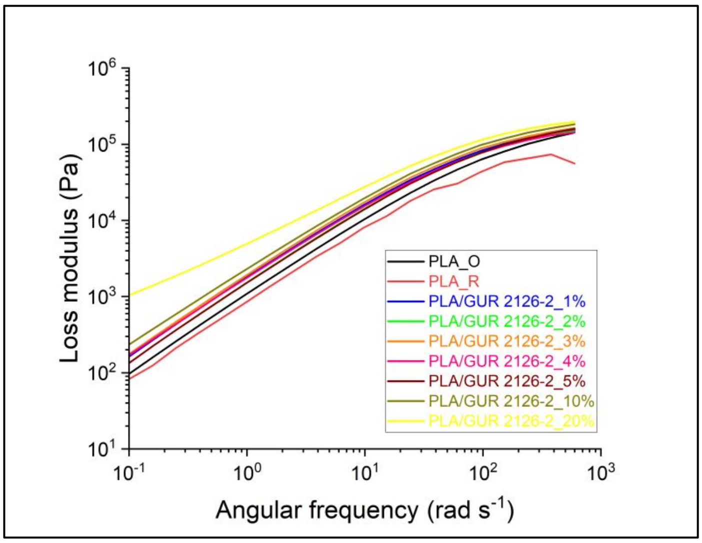

Fig. S 12. Rheological properties of PLA and PLA/GUR composites at GUR loading 0-20 wt% at 200 °C, a dependence of loss modulus on the angular frequency for PLA/GUR 2126-2.

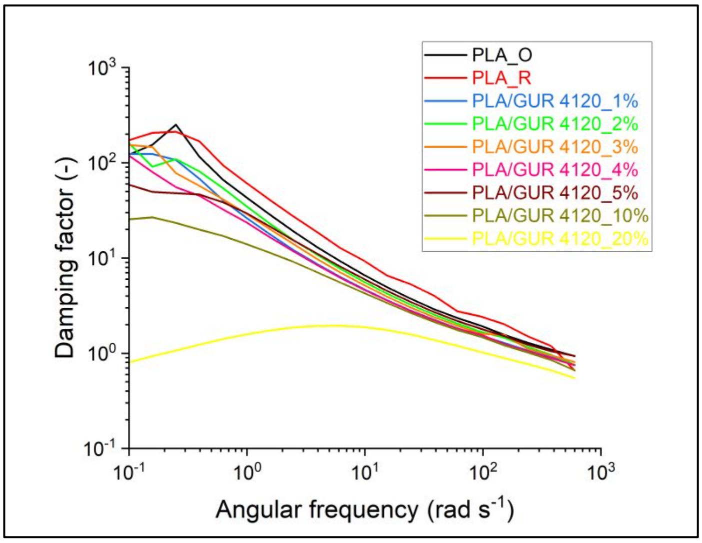

Fig. S 13. Rheological properties of PLA and PLA/GUR composites at GUR loading 0-20 wt% at 200 °C, a dependence of damping factor on the angular frequency for PLA/GUR 4120.

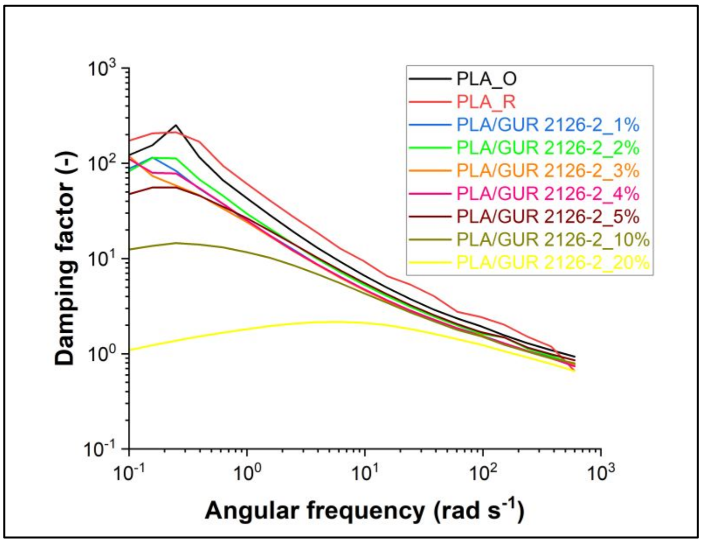

Fig. S 14. Rheological properties of PLA and PLA/GUR composites at GUR loading 0-20 wt% at 200 °C, a dependence of damping factor on the angular frequency for PLA/GUR 2126-2.

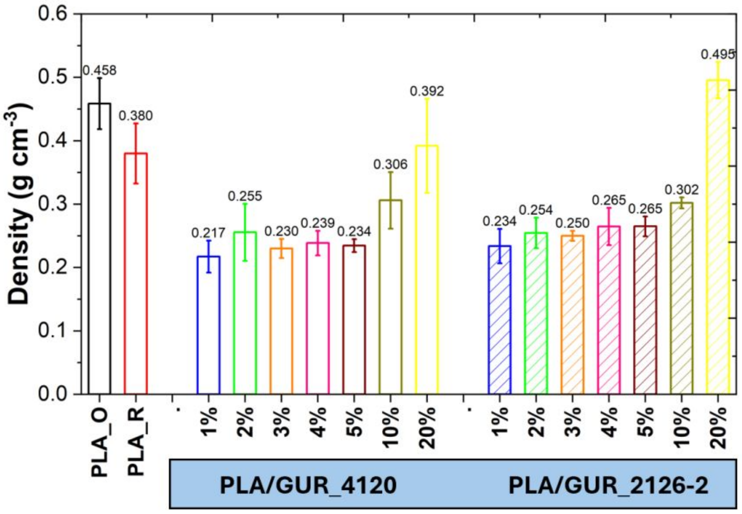

Fig. S 15. Density of PLA and PLA/GUR foams containing untreated GUR 4120 particles and hydrophilically surface-treated GUR 2126-2 particles at filler loadings 0-20 wt%.

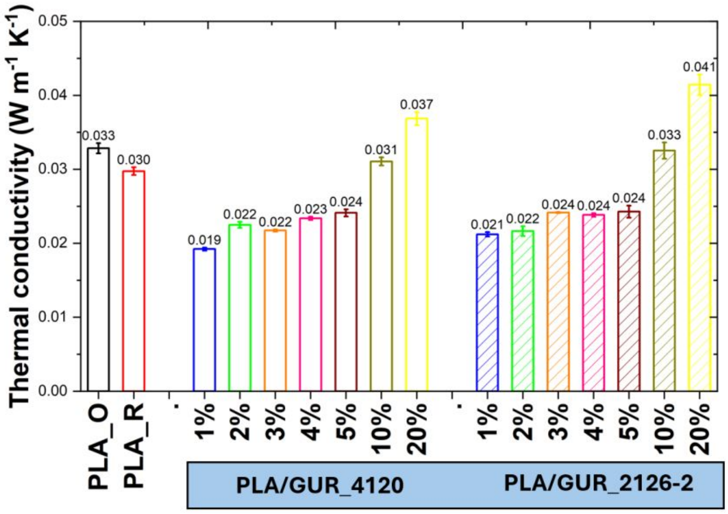

Fig. S 16. Thermal conductivity of PLA and PLA/GUR foams containing untreated GUR 4120 particles and hydrophilically surface-treated GUR 2126-2 particles at filler loadings 0-20 wt%.

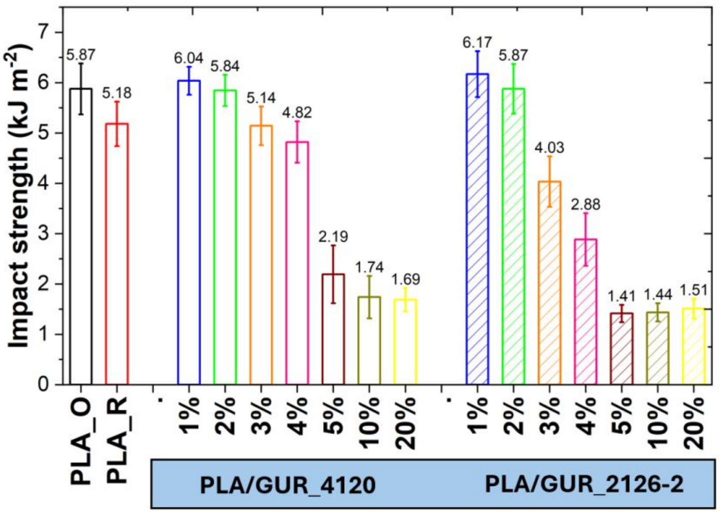

Fig. S 17. Impact strength of PLA and PLA/GUR foams containing untreated GUR 4120 particles and hydrophilically surface-treated GUR 2126-2 particles at filler loadings 0-20 wt%.

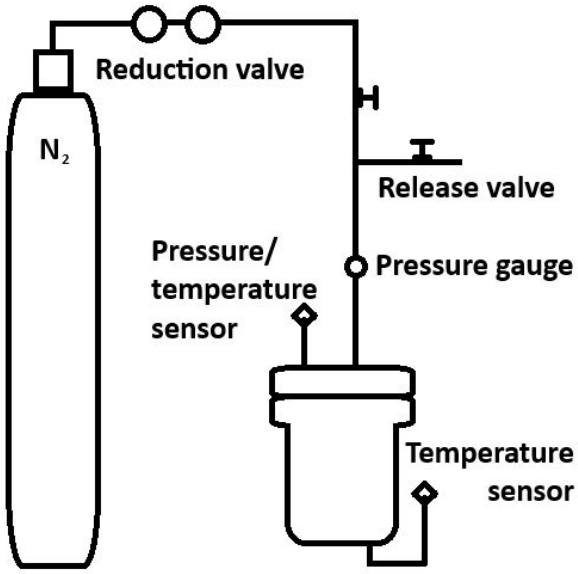

Fig. S 18. Schematic of the batch foaming apparatus: N_2 cylinder, autoclave chamber, and temperature/pressure controllers.

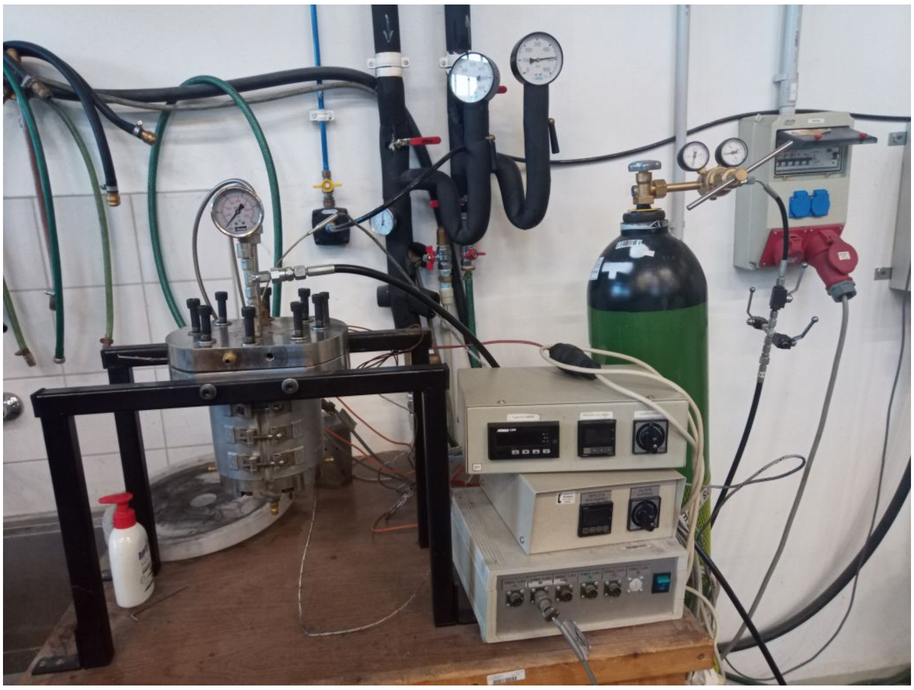

Fig. S 19. Batch foaming setup comprising an N_2 cylinder, an autoclave chamber, and temperature/pressure control units.

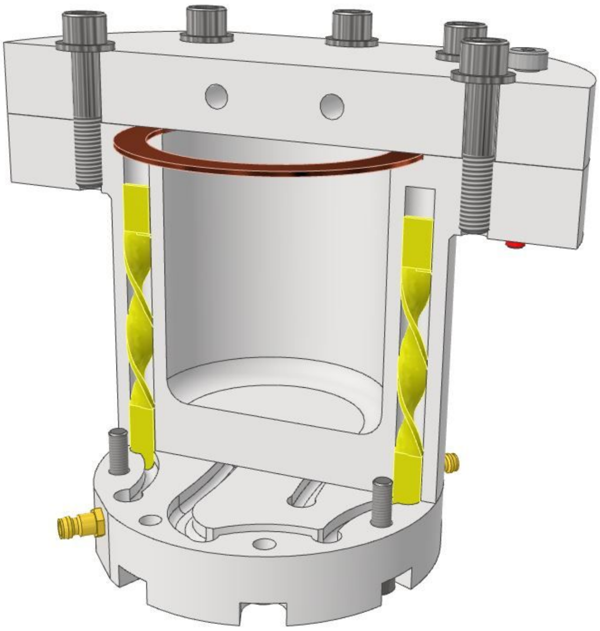

Fig. S 20. Schematic of the batch foaming chamber.
